# Development of a research agenda for medical grade footwear in the Netherlands: A multidisciplinary multiphase project to determine the key research questions to advance scientific knowledge in the field

**DOI:** 10.1002/jfa2.12016

**Published:** 2024-07-02

**Authors:** Jaap J. van Netten, Rutger Dahmen, Fred Holtkamp, Johanna P. Aussems, Gaston Jansen, Esther Mik, Sicco A. Bus

**Affiliations:** ^1^ Department of Rehabilitation Medicine Amsterdam UMC University of Amsterdam Amsterdam The Netherlands; ^2^ Amsterdam Movement Sciences, Rehabilitation & Development Amsterdam The Netherlands; ^3^ Amsterdam Rehabilitation Research Center/Reade Amsterdam The Netherlands; ^4^ Department of Allied Health Professions Fontys University of Applied Science Eindhoven The Netherlands; ^5^ Merem Medische Revalidatie Hilversum The Netherlands; ^6^ Loopvisie b.v. Lelystad The Netherlands; ^7^ Livit Orthopedie Amsterdam The Netherlands

**Keywords:** children, consensus development conferences, diabetes mellitus, foot deformities, foot diseases, foot ulcer, orthotic devices, rehabilitation, rheumatoid arthritis, shoes

## Abstract

**Background:**

The field of medical grade footwear is dynamic. Originally, a field where individual knowledge, expertise and skills determined the footwear and its outcomes, now becoming a more evidence‐based and data‐driven field with protocols and systems in place to create appropriate footwear. However, scientific evidence concerning medical grade footwear is still limited. Evidently, all stakeholders, from patients to pedorthists to rehabilitation physicians, will profit from a larger evidence‐base in this field. A widely supported research agenda is an essential first step to advance and facilitate new knowledge.

**Methods:**

We formed a multidisciplinary team and followed the methodology from Dutch medical societies for the development of a research agenda on medical grade footwear. This consisted of seven steps: (1) inventory of relevant questions with users and professionals; (2) analyses of responses; (3) analyses of existing knowledge and evidence; (4) formulating research questions; (5) prioritising research questions by users and professionals; (6) finalising the research agenda and (7) implementing the research agenda.

**Results:**

In phase 1, 109 participants completed a survey, including 50% pedorthists, 6% rehabilitation physicians and 3% users. Participants provided 228 potential research questions. In phases 2–4, these were condensed to 65 research questions. In phase 5, 152 participants prioritised these 65 research questions, including 50% pedorthists, 13% rehabilitation physicians and 9% users. In phase 6, the final research agenda was created, with 26 research questions, categorised based on the International Classification of Functioning Disability and Health ‘process description assistive devices’. In phase 7, an implementation meeting was held with over 50 stakeholders (including users and professionals), resulting in seven applications for research projects based on one or more research questions from the research agenda.

**Conclusions:**

This research agenda structures and guides knowledge development within the field of medical grade footwear in the Netherlands and elsewhere. We expect that this will help to stimulate the field to tackle the research questions prioritised and with that to advance scientific knowledge in this field.

**Abbreviations**
OFOM: Ontwikkelingsfonds Orthopedisch Maatschoenbedrijf (Dutch); in English: Development Fund Orthopaedic Footwear Companies

## 1 BACKGROUND

Medical grade footwear constitutes footwear that meets the specific medical needs of a person [1]. Medical grade footwear is a combination of the shoe and its insole and can be custom‐made or prefabricated [2]. It is also known under a variety of other terms, such as bespoke footwear, custom‐made footwear, orthopaedic footwear, pedorthic footwear or therapeutic footwear [2]. This footwear is prescribed for people with a wide range of diseases or disorders, such as diabetes mellitus, rheumatoid arthritis, degenerative foot disorders, muscle diseases and more [1]. The aim of medical grade footwear is to increase people's capacity for mobility, for example, by enhancing stability, supporting deformities, reducing pain, protecting from trauma or redistributing plantar pressure [1]. In the Netherlands alone, with a population of 18 million people, more than 100,000 pairs of medical grade footwear are prescribed and provided each year with total costs exceeding 200 million Euros [3].

Traditionally, medical grade footwear is made primarily based on the individual expertise, skills and experience of the pedorthist (also known as [orthopaedic] shoe technician) and prescriber (e.g., specialist in rehabilitation medicine, orthopaedic surgeon, rheumatologist etc.). Currently, a transition is taking place with the aim of delivering higher quality footwear, in which scientific‐based, data‐driven, automated and protocolled footwear solutions are used in the prescription and production processes [4, 5]. However, the transition is slow, scientific knowledge is still limited and the implementation of new and existing knowledge is hampered and fragmented due to a lack of structures to promote it. More scientific knowledge within the field of medical grade footwear is required to increase the value and quality of such footwear.

A first step in the development of more and higher‐quality scientific knowledge is having a widely supported research agenda. A research agenda consisting of relevant and feasible ideas for scientific research gives guidance to the field with respect to choices of how to use the available resources [6]. This guidance can then be used to undertake scientific research that is prioritised by both users (i.e., people who need medical grade footwear) and professionals (e.g., pedorthists, rehabilitation physicians, podiatrists and researchers) to help maximise the impact in daily practice.

Fields adjacent to medical grade footwear have developed research agendas (e.g., in diabetes‐related foot disease [7], in podiatry [8] or in foot and ankle osteoarthritis [9]), and several initiatives have been undertaken in the past to gather and prioritise relevant research questions in the field of medical grade footwear of which one has been published [10]. However, there is no widely supported research agenda specifically focussing on medical grade footwear, despite the importance and widespread use of this assistive device. Thus, as a result, scientific research and knowledge development in this field currently lack direction. Therefore, our aim was to develop a widely supported research agenda on medical grade footwear with input from all stakeholders.

## 2 METHODS

### 2.1 Design and setting

We followed the design for multidisciplinary research agenda development as outlined by the Dutch Federation of Medical Specialists Knowledge Institute [6, 11] and the Netherlands Organisation for Health Research and Development (“ZonMw”) [12]. This methodology consists of seven phases described in detail in the next sections.

The Development Fund for Orthopaedic Footwear Companies (in Dutch: Ontwikkelingsfonds Orthopedisch Maatschoenbedrijf (the OFOM Foundation)) provided the funding for this project following an idea from one of its board members (SB). The funder then invited this board member to chair (SB) the project, and—together with the project manager (JvN)—to set up a multidisciplinary working group to execute the project. Two working group members were also board members of the OFOM Foundation (SB and RV; see declaration of interest); their working group actions were independent of the OFOM Foundation. The OFOM Foundation had no involvement or authorisation over the process, the results and the decision to publish. The chair and project manager invited a multidisciplinary working group consisting of 10 members (the seven authors of this paper and the three members specified in the acknowledgments) who all accepted their invitation. The setting for the research agenda was the Netherlands.

### 2.2 Participants

Participants were included in phases 1 and 5. Other phases were executed by the 10 members of the multidisciplinary working group. Regarding professional background, these were human movement scientists (*n* = 2), pedorthists (*n* = 3), rehabilitation physicians (*n* = 2), lecturer/researcher in orthopaedic technology (*n* = 1), manager of an orthopaedic devices company (*n* = 1) and a health insurer (*n* = 1).

### 2.3 Phases of research agenda development

The process of developing the research agenda consisted of seven phases. At the first working group meeting, this process was explained, discussed and established.

#### 2.3.1 Phase 1: Inventory of potential research questions by stakeholders

To perform an inventory of potential research questions by stakeholders, a short survey was prepared by the working group to identify research questions from stakeholders in medical grade footwear. Research questions were defined as “questions in the field of medical grade footwear that can be answered by scientific research”. This survey consisted of five questions to establish participants' background characteristics, and free fields where up to five research questions could be added, with additional room to provide background with the proposed research question. We used the online program LimeSurvey (Open‐source application; www.limesurvey.org), and the survey could be completed between March 26 and April 18, 2021. The survey was widely distributed in the field in the Netherlands, through newsletters from various organisations, personal emails to colleagues, clients and patients, and through social media.

An explanation of the project and an informed consent button covered the front page. Participants had to provide informed consent before they could start with the survey. The research was exempt from formal medical ethical approval under Dutch law. The research methodology was checked and approved by the privacy officer of Amsterdam UMC.

#### 2.3.2 Phase 2: Analysing outcomes of the inventory

The outcomes of the inventory were analysed by the working group members. During the second working group meeting, the working group identified five domains into which potential research questions from phase 1 could be classified: Behaviour and user (defined as: research questions concerning footwear use and user experiences); clinical effectiveness (research questions that investigate medical grade footwear with primarily a clinical outcome measure); technical effectiveness (research questions that investigate medical grade footwear with primarily a biomechanical outcome measure); processes (research questions concerning the processes of prescribing, fitting or delivering medical grade footwear); and, innovations (research questions concerning the development of new products or services). All questions were then categorised into a domain by two authors (JvN and SB). Within each domain, research questions were clustered. If a question could not be answered through scientific research or could not be operationalised as a research question, it was excluded. For each domain, all these steps were independently checked by at least three additional working group members to assess whether the merging and formulation had been performed adequately and carefully. Working group members could make clarifications, could delete questions for lack of relevance to daily practice and could also add research questions.

In parallel, one‐on‐one in‐depth interviews were held by one of the working group members (JvN) with six experts from the field (one user, three pedorthists, one physician/scientist and one with a commercial position in a footwear company) to discuss the results of the initial inventory. They were asked whether the domains and research questions were relevant and well formulated, and whether in their opinion any research questions were missing.

#### 2.3.3 Phase 3: Review and analysis of peer‐reviewed scientific literature

In phase 3, the available scientific literature was reviewed and analysed for the purposes of: (i) assessing if research questions listed had already been answered and (ii) assessing if additional relevant research questions were formulated in research publications. We searched the literature in May and June 2021 for systematic reviews related to the topic (Additional File 2).

#### 2.3.4 Phase 4: Formulating research questions for the research agenda

In phase 4, a list of research questions was formulated with the aim of using the list for priority assessment by stakeholders. The list of research questions from phase 2 was taken as starting point and combined with the findings from the literature review in phase 3. Furthermore, the list had to be suitable for priority assessment. We defined this as a list that could be read in full within 20 min (assuming a reading speed of 150 words per minute), and that each research question should be understandable without need for further explanation. At the third working group meeting, these steps were discussed until consensus was reached on the content and wording of all research questions.

#### 2.3.5 Phase 5: Prioritisation of research questions by stakeholders

To prioritise the research questions, another survey was created in LimeSurvey using the same methods as in Phase 1. For each of the five domains, the research questions were listed in random order, and respondents were asked to select up to a maximum of three research questions per domain that they felt were priorities. In the priority setting, participants were asked to consider the relevance, urgency, impact and feasibility of the research question. The survey was available from July 6 to August 9, 2021. The survey was widely distributed in the Netherlands, in the same way as the survey in Phase 1, through newsletters from various professional organisations, personal emails to colleagues and users, and through social media. Users were also approached through working group members who interacted with users in their daily work. In addition, users were also offered the option of completing the survey on paper to facilitate readability, which was then processed digitally by entering the responses into LimeSurvey.

#### 2.3.6 Phase 6: Finalising the research agenda

At the fourth working group meeting, all results of the prioritisation survey were discussed. The original purpose of this discussion was to formulate a research agenda consisting of the top‐10 research questions, taking into account the priority as given to the different domains and research questions. However, the working group concluded unanimously that no clear top 10 could be identified. As a result, a different methodology was required to finalise the research agenda. For this, the working group used the theoretical framework based on the Process Description of Assistive Devices [13] founded on the International Classification of Functioning, Disability and Health [14]. All research questions were re‐ordered based on the steps from the Process Description by one working group member (JvN) and checked by the other working group members. During the fifth working group meeting, the priority scores were discussed based on this new ordering.

#### 2.3.7 Phase 7: Implementing the research agenda

The working group reconvened for their sixth and seventh meeting to further facilitate implementation of the research agenda. The working group provided suggestions for projects related to the research agenda, including methodology, finances and project partners required, in an implementation document and organised an implementation meeting.

## 3 RESULTS

**FIGURE 1:**
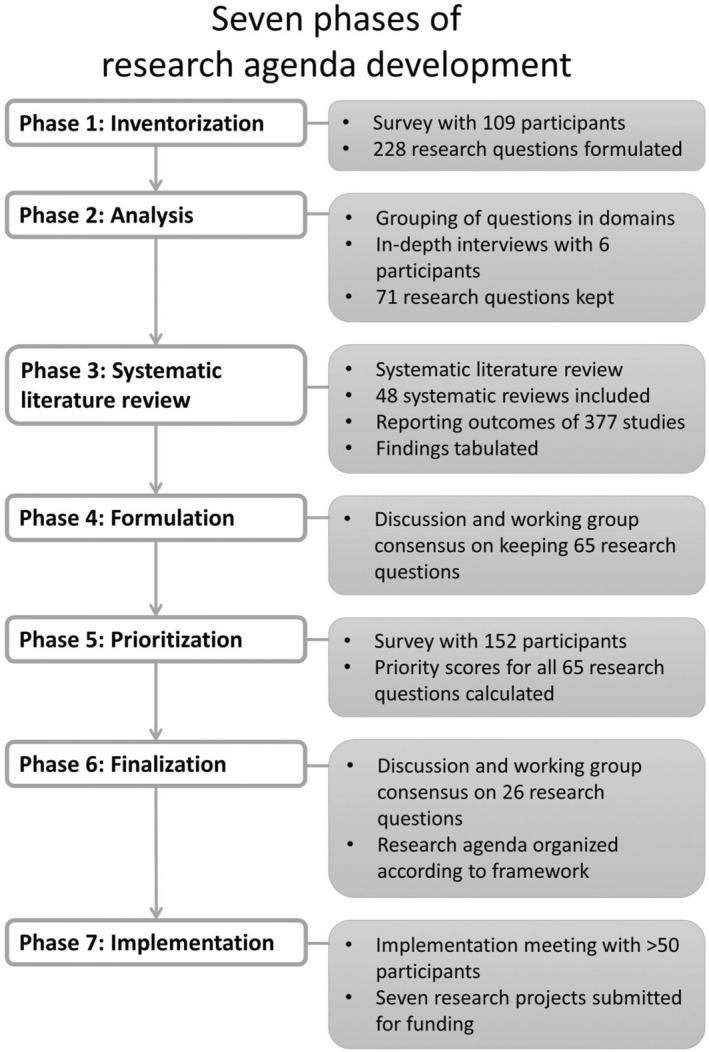
The seven phases in the development of the medical grade footwear research agenda.

The results in each of the seven phases of research agenda development are shown in Figure 1.

### 3.1 Phase 1: Inventory of potential research questions by stakeholders

The online survey page was accessed 253 times during the period it was open, informed consent was given 211 times and 109 participants completed the survey in full (Table 1). About half of the participants were pedorthists or similar, and most of them had more than 20 years of experience with providing medical grade footwear. A total of 228 questions were provided by the 109 participants. The average number of valid survey questions per respondent was 2.1 (standard deviation 1.5; minimum 0 and maximum 5). The complete list of 228 questions can be found in Additional File 1. All questions were formulated in Dutch. We used the neural machine translation service DeepL Translator (DeepL SE, Cologne, Germany) to unilaterally translate these research questions from Dutch to English with the resultant translation provided in Additional File 1.

**TABLE 1:** Demographics of participants in phases 1 (*n* = 109) and 5 (*n* = 152).

Feature	Category	Phase 1	Phase 5
Gender	Female	39% (*n* = 43)	44% (*n* = 67)
	Male	61% (*n* = 66)	56% (*n* = 85)
Age (years)	18–30	8% (*n* = 9)	16% (*n* = 24)
	31–40	23% (*n* = 25)	21% (*n* = 32)
	41–50	27% (*n* = 29)	26% (*n* = 40)
	51–60	31% (*n* = 34)	21% (*n* = 32)
	61–70	11% (*n* = 12)	13% (*n* = 20)
	71–80	0% (*n* = 0)	2% (*n* = 3)
	81 or older	0% (*n* = 0)	1% (*n* = 1)
Personal/professional background	User	3% (*n* = 3)	9% (*n* = 14)
	Pedorthist or similar technician	50% (*n* = 54)	50% (*n* = 76)
	Podiatrist	16% (*n* = 17)	10% (*n* = 15)
	Commercial position in footwear technology	6% (*n* = 7)	3% (*n* = 4)
	Physician	6% (*n* = 7)	13% (*n* = 19)
	Scientist	3% (*n* = 3)	7% (*n* = 11)
	Lecturer in footwear technology	5% (*n* = 5)	3% (*n* = 5)
	Health insurance company employee	5% (*n* = 5)	3% (*n* = 4)
	Other	7% (*n* = 8)	3% (*n* = 4)
Experience with medical grade footwear (years)	0–5	12% (*n* = 13)	20% (*n* = 30)
	6–10	10% (*n* = 11)	17% (*n* = 26)
	11–15	15% (*n* = 16)	16% (*n* = 25)
	16–20	10% (*n* = 11)	13% (*n* = 19)
	21 or more	53% (*n* = 58)	34% (*n* = 52)

### 3.2 Phase 2: Analysing outcomes of the inventory

Based on input from all working group members and the interviews, the list of research questions from phase 1 was modified. This resulted in a total 71 research questions in the five domains: behaviour and user: *n* = 10; clinical effectiveness: *n* = 15; technical effectiveness: *n* = 15; processes: *n* = 14 and innovations: *n* = 17.

### 3.3 Phase 3: Review and analysis of peer‐reviewed scientific literature

We included 48 systematic reviews, reporting outcomes from a total 377 studies (Additional File 2). We found that most studies investigated the effects of footwear fitting on kinetics/kinematics and pain in osteoarthritis (60 studies) and on plantar pressure in people with diabetes‐related neuropathy (57 studies). In people with diabetes‐related neuropathy, only 11 studies were available on the primary clinical outcome (foot ulceration). Research on the effects of medical grade footwear in children was less frequent compared to research in adults (41 systematic reviews on adult populations and 7 on paediatric). While most systematic reviews concluded that positive effects were found from medical grade footwear, they also universally concluded that more research is needed. This especially concerned research of higher quality, with adequate design, control conditions and follow‐up. These are always dependent on the exact research question, but in general, there is a need for studies with a prospective design, with a control group that is treated at the same time and according to standard clinical practice, with follow‐up that is long enough to observe clinical outcomes and with outcome assessors blinded for the treatment provided. For studies where a cross‐sectional design may suffice, the comparator condition(s) should be carefully chosen: this should include a footwear condition that is standard practice. For the research agenda, this means that the final research questions selected should be answerable by means of high‐quality research with limited risk of bias.

### 3.4 Phase 4: Formulating research questions for the research agenda

Based on the activities within phase 4, 65 research questions were formulated in the five domains (behaviour and user: *n* = 11; clinical effectiveness: *n* = 11; technical effectiveness: *n* = 14; processes: *n* = 14 and innovations: *n* = 15). These research questions are provided in Additional File 3 translated from Dutch to English using DeepL Translator (see Phase 1 for details).

### 3.5 Phase 5: Prioritisation of research questions by stakeholders

The survey was opened 199 times during the period it was open, and permission to collect data was given 178 times. A total 152 participants completed the survey completely for at least 1 domain, of which 132 people completed all steps of the survey. All surveys where at least 1 domain was fully completed were included. Descriptive data of the 152 participants shows that about half of the respondents were pedorthists or similar, about one‐third of the respondents had more than 20 years of experience with medical grade footwear and characteristics were relatively similar to the first survey (Table 1).

In the results of the survey, we found that all 65 research questions were chosen as priority by at least 6% of respondents. There were 19 research questions prioritised by 30% or more of respondents and 1 question by 40% or more. All scores for each research question, classified by domain and in order of highest to lowest priority frequency, are shown in Additional File 3. In addition to prioritising research questions within each domain, participants were also given the opportunity to prioritise the domains. No domain was clearly getting prioritised over others (Table 2, statistical analyses deemed not appropriate given the exploratory nature).

**TABLE 2:** Prioritisation of the five domains within the research agenda in phase 5.

Domain	All respondents (*n* = 133)	Professionals (*n* = 120)	Users (*n* = 13)
Behaviour and user	28% (*n* = 37)	27% (*n* = 32)	39% (*n* = 5)
Clinical effectiveness	18% (*n* = 24)	20% (*n* = 24)	0% (*n* = 0)
Technical effectiveness	17% (*n* = 23)	18% (*n* = 21)	15% (*n* = 2)
Processes in foot care	8% (*n* = 11)	8% (*n* = 9)	15% (*n* = 2)
Innovation	22% (*n* = 29)	23% (*n* = 28)	8% (*n* = 1)
All domains are equal	7% (*n* = 9)	5% (*n* = 6)	23% (*n* = 3)

*Note*: Numbers show the number of participants that indicated this domain as having the highest priority.

### 3.6 Phase 6: Finalising the research agenda

**FIGURE 2:**
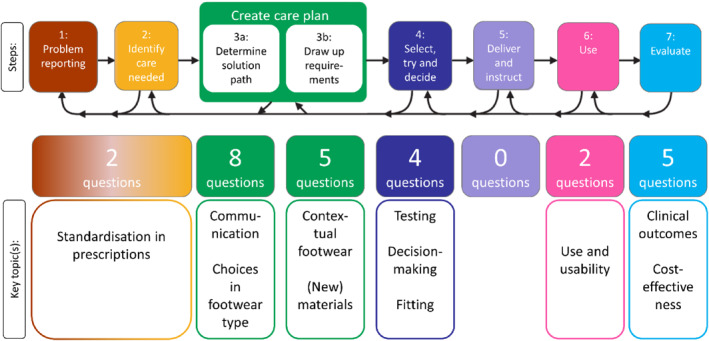
Number of questions included in the final research agenda, mapped according to the theoretical framework [13], with key topics described per step of the framework.

The theoretical framework used for the research agenda, the Process Description of Assistive Devices [13], is organised in seven steps (Figure 2; Table 3). The 26 research questions included in the research agenda and their priority scores from phase 5 can be found in Table 3 organised in accordance with this framework. The working group judged, based on the results from phases 1–5, that research questions in domains 3a (determining the solution), 6 (use) and 7 (evaluate) are of the highest priority.

**TABLE 3:** The research questions included in the final research agenda and their priority scores.

Steps of the process description (shaded) and research questions (numbered)	Priority score		
	All (*n* = 152)	Prof. (*n* = 138)	Users (*n* = 14)
Steps 1 and 2: Problem identification and request for care			
1. To what extent can the request for care or medical need(s) and the foot‐related diagnosis be made (more) objective, and does that lead to more uniformity in footwear prescription?	27%	29%	15%
2. To what extent can the complexity of the request for care related to medical grade footwear be expressed in a single score?	16%	14%	31%
Step 3a: Plan of care—determining the solution			
3. What are the expectations and preferences of people who are prescribed with medical grade footwear, and to what extent can these be kept realistic?	34%	34%	29%
4. To what extent can the acceptance of medical grade footwear be promoted in people who struggle with this acceptance?	35%	36%	21%
5. To what extent can the communication with and education of the user during the prescription and delivery of medical grade footwear be improved, and does this lead to more use and better usability?	32%	33%	14%
6. To what extent can the appearance of medical grade footwear be digitally visualised prior to footwear provision, and does that lead to better expectations, acceptance and use?	30%	34%	0%
7. Which specific foot conditions or diagnoses fit with a specific type of medical grade footwear, that is, prefabricated, semi custom‐made, fully custom‐made and under what conditions do you switch to another type?	36%	39%	8%
8. Does multidisciplinary over single‐disciplinary provision of medical grade footwear lead to more use and better usability?	36%	37%	21%
9. What is the cost‐effectiveness of temporary medical grade footwear compared to total contact casts in the treatment of plantar diabetes‐related foot ulcers?	36%	37%	21%
10. What is the most appropriate moment to move from a total contact cast or other offloading treatment for a plantar diabetes‐related foot ulcer to medical grade footwear?	36%	37%	23%
Step 3b: Plan of care—program of requirements			
11. To what extent can medical grade footwear specifically for summer season conditions be developed that has similar effectiveness as regular medical grade footwear but is better designed to ventilate under hot and sweaty feet conditions?	33%	29%	70%
12. Does having different pairs of medical grade footwear for different (social) situations result in more use and better usability?	26%	25%	36%
13. To what extent can new materials or devices be used to make lighter‐weight but equally effective medical grade footwear?	33%	33%	31%
14. What factors determine the wear, tear and life span of medical grade footwear?	20%	16%	62%
15. What is the use and what are the effects of using new materials and design and manufacturing techniques for medical grade footwear (e.g. 3D printing) compared to more traditional materials and techniques?	22%	22%	15%
Step 4: Select, try and decide			
16. What is the effect of first providing a person with temporary medical grade footwear, followed by definitive medical grade footwear, in comparison with directly providing definitive medical grade footwear, on use, biomechanics and usability of the definitive medical grade footwear?	33%	35%	15%
17. To what extent can shared decision‐making be implemented in the provision of medical grade footwear, and does it lead to more use and better usability?	21%	20%	29%
18. What is the difference between digital fitting and fitting with plaster cast on the effectiveness, usability and costs of medical grade footwear?	33%	34%	15%
19. What is difference between fitting under loaded or unloaded conditions for medical grade footwear on the biomechanical effectiveness of the footwear?	31%	34%	7%
Step 5: Deliver and instruct			
No research questions prioritised in this domain.			
Step 6: Use			
20. What factors influence the use and usability of medical grade footwear?	37%	36%	50%
21. What is the usability of medical grade footwear, and how does it change during long‐term use?	15%	13%	36%
Step 7: Evaluate			
22. What are the appropriate uniform clinical and patient‐reported outcome measures for determining and monitoring the effectiveness of medical grade footwear nationwide?	39%	38%	57%
23. What is the cost‐effectiveness of using in‐shoe plantar pressure measurement for evaluating and improving medical grade footwear in people with sensory loss but without a previous plantar foot ulcer?	50%	48%	64%
24. What is the effect of wearing medical grade footwear on muscle strength in the foot and lower leg?	32%	30%	50%
25. What is the effectiveness of medical grade footwear in people at increased risk of falling?	32%	32%	36%
26. What is the effectiveness of medical grade footwear in children? *	22%	24%	0%

*Note*: Here, the research questions described were translated from Dutch to English using DeepL (see details in the methods) and subsequently modified and improved from a language perspective by the authors. The priority score indicates the percentage of respondents that selected this research question as having priority in phase 5. Prof. = professionals (e.g., pedorthists and physicians; see Table 1 for a full list) *: This question was not formulated as such in the priority phase, but actually consisted of four specific research questions related to children (see Table 5 in Additional File 3); the question was reformulated after the priority setting phase by the working group, while the priority scores provided are taken from the research question with the highest priority score that concerned both the children and the medical grade footwear; see further the specific section on the discussion for the rationale behind this formulation.

### 3.7 Phase 7: Implementing the research agenda

Following the decisions in phase 6, the final research agenda was written in Dutch. This document was publicly presented on December 2, 2022, during the Annual Meeting of the Dutch Branch Organisation for Orthopaedic Shoe Technicians (“NVOS‐Orthobanda”) and published (in Dutch) on the website of the OFOM Foundation [15].

Subsequently, an implementation document was written and widely shared, using the same strategies as in phases 1 and 5, together with an invitation for an open implementation meeting. The implementation meeting was held on June 26, 2023. During that meeting, seven researchers pitched a project idea to answer one or more research questions from the research agenda. Open discussions were then held between the researchers and the 50+ stakeholders present (pedorthists, podiatrists, rehabilitation physicians, researcher and patient representatives; exact numbers are not available), to stimulate co‐creation and subsequent multidisciplinary collaborations. Based on these discussions, the researchers are currently setting up projects and writing grant applications. The funder and initiator of the research agenda (the OFOM Foundation) opened a specific call and funds for these research projects, while other grant agencies are also targeted. The applications for these projects are—at the moment of writing this publication—ongoing.

## 4 DISCUSSION

The research agenda for medical grade footwear presented here is the first such research agenda in this field of assistive devices. Medical grade footwear supports millions of people worldwide with a variety of diseases and disorders and is an industry that is large in numbers and finances [1]. Despite this, systematic knowledge development through scientific research lags behind other fields. For this research agenda, we followed an extensive methodology for research agenda development [6] going through multiple phases with multidisciplinary input from all stakeholders, including users. In this discussion, we reflect on the process as a whole and on the research questions prioritised.

In order to arrive at a widely supported research agenda, a multidisciplinary working group was set up, and stakeholders in the field were given the opportunity to provide input through two broad surveys, extended with in‐depth interviews. The importance of including different perspectives can be recognised in the results in phase 5: different people have different priorities, as all 65 research questions were prioritised by ≥6% of respondents, 19 different research questions were prioritised by ≥30%, and only one by ≥40%. We specifically assessed differences in priorities between professionals and users. We found agreement on most priorities, but users gave higher priority on some research questions, for example, related to the development of orthopaedic footwear for the warmer summer season, research on wear and tear and on development of a general set of outcome measures (see Table 3). This underlines the importance to include users' perspectives in future projects.

In the final research agenda, priority was given to a broad variety of research questions. These questions cover all steps in the process of prescription of medical grade footwear, highlighting the limited scientific evidence generally found in this field. Of the questions in which a specific population was mentioned, questions concerning people with diabetes were remarkably frequently prioritised. This may be explained by the large prevalence and disease burden of people with diabetes who require medical grade footwear increasing the need for high‐impact research. It may also reflect that many healthcare providers work with this population, probably also within the respondents, hence resulting in more frequent prioritisation. However, we have no further insights into the respondent's background and experiences, as— partly for privacy reasons—the survey did not collect this information.

The decision to use the Process Description of Assistive Devices [13] as framework for the research agenda was not taken a priori. This deviation was necessary because a short “top‐10 research priorities” (such as others have created [7]) did not emerge from the priority setting phase. Choosing this framework, widely used in daily clinical practice in the Netherlands, created more clarity in terms of the interrelationship between the various research questions in the final research agenda. This facilitates setting up projects tackling multiple research questions at once. Therefore, we will discuss the research questions within each step of this framework (see Table 3 for all the research questions and their number).

### 4.1 Steps 1 and 2: Problem identification and requests for care

The two questions formulated within steps 1 and 2 reflect the wish to create more standardisation in a field where ‘custom‐made’ also induces the risk of a lack of uniformity. Having an index score for complexity underlies the perceived challenges by professionals that some users, pathologies or conditions are much harder to treat successfully compared to others, while this challenge is generally not reflected in reimbursements for provided footwear.

### 4.2 Step 3: Care planning

Half of all research questions in the research agenda concern step 3. Step 3 involves the key step of care planning and is a combination of determining the solution (step 3a) and creating a program of requirements (step 3b). Questions 3–6 follow from earlier observational research finding associations between expectations, acceptance and communication with footwear adherence [16, 17, 18, 19]. However, there is limited research regarding interventions to target these factors [16], and this next step is outlined in the research questions in the research agenda.

Questions 7–10 reflect a limitation in the available evidence. Existing studies generally focus on one specific type of medical grade footwear, yet an important question in daily clinical practice concerns the choice between the various types available, such as between prescribing prefabricated, semi custom‐made or fully custom‐made footwear. In addition, questions 9 and 10 reflect the importance of footwear in the diabetes population and the disparity between guidelines (recommending casts [20]) and clinical practice (frequently treating these ulcers with footwear [21, 22, 23, 24]). The one study directly comparing casts with custom‐made footwear as provided in the Netherlands is rather old and not reflective of current day medical grade footwear [25]; hence, the selection of this research question is to stimulate new research on this topic.

Questions 11 and 12 reflect a priority in the development of contextual footwear, whether specifically for the summer or in general. Recent studies on the development of specific medical grade footwear for inside the house shows the opportunities in improving user experiences and wearing adherence by providing footwear for different contexts—in this case for inside and outside the house [26, 27]. We hope that the positive outcomes from such “indoor footwear”, together with the priorities indicated here, inspire others to also develop and investigate more types of contextual footwear. Questions 13–15 all concern material characteristics, and reflect a path that has only been explored to a limited extent for medical grade footwear [28, 29].

### 4.3 Step 4: Select, try and decide

In step 4 of the Process Description, user and professional are expected to select the most optimal assistive device, to try this, and to make decisions. Question 16 reflects the opportunity to facilitate this process by first creating so‐called ‘temporary orthopaedic footwear’. A solution frequently used in daily clinical practice in the Netherlands yet never investigated. Question 17 reflects the decision‐making process. Shared‐decision making is well‐known in medicine but never studied in relation to medical grade footwear [16].

Another aspect that plays a role in this step in the process is the method with which the footwear is made. While some research on this topic is available, this primarily focuses on process outcomes, such as difference in time, accuracy and reliability of digital fitting versus other techniques [30]. From a user‐perspective, it is more important to investigate effectiveness and usability outcomes.

### 4.4 Step 5: Deliver and instruct

No research questions were formulated in this step.

### 4.5 Step 6: Use

Use of medical grade footwear has been investigated for decades (e.g., as summarised here [31] and here [32]). Despite these studies, this research question is considered relatively unanswered seeing the high priority scores. Concerning factors associated with use, explained variance in most studies is low [31], already pointing at the necessity for more research. Concerning use itself, we think that the relevance of the research question can be explained by a number of factors. First is the ever‐changing reality, where studies performed >10 years ago (e.g., [19, 33]) may not be reflective of the current day practice anymore, thanks to improvements made in footwear prescription and creation. Second, most studies measured ‘footwear use’ based on participant's self‐report of their use or adherence; however, measuring ‘footwear use’ via self‐report is not reliable as recently proven [34]. Third, use of medical grade footwear is not a static measure, yet existing studies measure only short periods of time [35, 36, 37]. Fourth and finally, most studies only investigated footwear use in people with diabetes at a risk of foot ulceration, rather than the broad population that is provided with medical grade footwear. For all these reasons, research into use and usability of medical grade footwear remains a priority.

### 4.6 Step 7: Evaluate

An important finding from the literature review was the need for more and higher quality research into the effects of medical grade footwear, as there is still (too) much unknown about their outcomes. Before such studies are undertaken, research question 22 should perhaps be answered at first, as there are no widely agreed clinical or patient‐reported outcome measures in this field. Other fields have shown the methodology to do this, such as within OMERACT (https://omeract.org).

Question 23 stresses the importance of investigating the cost‐effectiveness of in‐shoe pressure measurements. These measurements are getting implemented more widely globally [38, 39, 40], yet the only interventional study is in one specific population [41]. Question 24 concerns the effects of medical grade footwear on muscle strength. This has been investigated in healthy populations but not in those with diseases or disorders [42]. Such research is required, as medical grade footwear may increase muscle strength when people regain their activity thanks to the footwear, but it may also reduce strength as a result of the footwear taking over some walking aspects that require foot muscle activation. The same contradiction can be seen for question 25, regarding falling. Footwear can be preventative, such as a shown in a recent study on a podiatric intervention (including the prescription of proper, but not medical grade, footwear) [43]. But, footwear might also induce falling, if, for example, a rocker is chosen that reduces pressure and at the same time decreases stability [44].

Finally, research question 26 was formulated rather broadly. The survey contained several specific questions around the effect of medical grade footwear for children, but these scored low in terms of prioritisation. This perhaps reflects the smaller number of healthcare professionals involved in the surveys that are specialised in working with children. However, with the scarce research in this population and the importance for the children who are provided with it, we decided as the working group against excluding all these specific research questions based on low priority scores. As an alternative, we decided to include this broad question in the final research agenda to serve as starting point for researchers and clinicians to develop more specific research questions that may fall under this umbrella question. With this, we hope to stimulate further research on medical grade footwear in paediatric populations.

### 4.7 Strengths and limitations

A strength of this project was the extensive methodology followed. We used existing, and widely‐used, methodology for the development of research agendas [6]. This methodology includes multiple phases where input is obtained from a large variety of stakeholders, including users as well as assessment of the published scientific literature. We also invested in an open atmosphere within the working group, to ensure that all participants were able to phrase their—sometimes differing—opinions. This resulted in lively and interesting discussions within the working group. These various aspects are reflected in the broad research agenda presented here.

Given the lack of previous research agendas or similar projects in this field, a limitation was not defining sample sizes for our surveys in advance. With 109 respondents in phase 1 and 152 in phase 5, the working group was generally satisfied with the response. Industry estimates suggest a total 2100 pedorthists working in the Netherlands [45], which imply a response rate of 4% (*n* = 75 pedorthists in phase 5). Working group members suggested that 50–75 persons is the number of pedorthists generally showing up at conferences and meetings. Therefore, we think that we have captured the majority of the most active voices in the field, and with >100 responses on both surveys, we succeeded in capturing a diversity of voices.

Another limitation was the number of users who actively participated in the surveys, this was small with *n* = 9 and *n* = 14 for phases 1 and 5, respectively. There is no well‐trained user representation society for people with medical grade footwear in the Netherlands (or anywhere globally), which limits their participation in a complex undertaking, such as determining a research agenda. We experienced that the users who did participate struggled with the concept of prioritising research questions, partly explaining the relatively low participation numbers. We solved this by supporting them during the survey completion. To enhance user participation in similar projects for the future, steps are being taken to set up the first‐ever formal user representation group in this field.

A third limitation was the literature search in phase 3. We did not have the capacity to perform a full systematic review of all the peer‐reviewed medical literature concerning medical grade footwear. As an alternative, we searched for systematic reviews on the topic. We identified 48 systematic reviews reporting outcomes of a collective 377 studies. While these can be considered to provide an adequate picture of the evidence and study quality in the field, the findings are not exhaustive. Individual studies not included in a systematic review have been missed by design, while limiting the search to only one person who screened and assessed all records may have also led to potentially missing some publications. The key aim of this phase was to ensure no research questions would be included that have already been answered. To overcome the limitations described here, we informally searched the literature for each research question included in the final research agenda, thereby ensuring that none of them had already been answered.

Finally, this research agenda was developed for the Dutch situation with input from people working or living in the Netherlands. However, most research questions in the final research agenda are not situation‐dependent nor specific to the organisation of care. Therefore, we think that the research questions prioritised in this research agenda are representative for the global field of medical grade footwear, and we hope that this research agenda will also stimulate further research on this topic in other regions of the world.

## 5 CONCLUSIONS

This research agenda structures and guides knowledge development within the field of medical grade footwear in the Netherlands and elsewhere. We expect that this will help to stimulate the field to tackle the research questions prioritised and with that to advance scientific knowledge in this field. Important for implementing this research agenda is to maintain a multidisciplinary representation within research projects, and to strive for high‐quality scientific research with low risk of bias, that can be implemented when successful.

## AUTHOR CONTRIBUTIONS

**Jaap J. van Netten**: Conceptualization; formal analysis; funding acquisition; methodology; project administration; writing – original draft; writing – review & editing. **Rutger Dahmen**: Formal analysis; writing – review & editing. **Fred Holtkamp**: Formal analysis; writing – review & editing. **Johanna P. Aussems**: Formal analysis; writing – review & editing. **Gaston Jansen**: Formal analysis; writing – review & editing. **Esther Mik**: Formal analysis; writing – review & editing. **Sicco A. Bus**: Conceptualization; formal analysis; Funding acquisition; methodology; writing – review & editing.

## CONFLICT OF INTEREST STATEMENT

SB declares that he is board member of the OFOM Foundation, funder of this project. The other authors declare no competing interests. However, all authors do acknowledge their academic and professional interest in advancing the field of medical grade footwear and as such their interest in this research agenda.

## ETHICS STATEMENT

Ethics approval was not required in accordance with Dutch law. All participants provided written informed consent before participating.

## Supporting information

Supporting Information S1

Supporting Information S2

Supporting Information S3

## Data Availability

All data generated or analysed during this study are included in this published article and its supplementary information files.
