# Supplementary material for: Development of a research agenda for medical grade footwear in the Netherlands: A multidisciplinary multiphase project to determine the key research questions to advance scientific knowledge in the field
Source: J Foot Ankle Res. 2024 Jul 2;17(3):e12016. doi: 10.1002/jfa2.12016 (PMC11633342; doi:10.1002/jfa2.12016)
Supplement: Supplementary file 1 — Supporting Information S1 [file JFA2-17-e12016-s001.pdf]

Additional File 1 – part of the manuscript “Development of a research agenda for medical grade footwear: a multidisciplinary multiphase project to determine the key research questions to advance scientific knowledge in the field”

Additional file 1: Complete list of research questions as suggested by stakeholders in the first survey (Phase 1)

The table below shows the full list of 228 research questions as provided by the 109 individuals who completed the initial survey in full. The research questions as described in this file are the raw data, that is, no edits or changes have been made to the texts below. The research questions are arranged alphabetically, based on the first word in the "research question" column.

The questions have been unilaterally translated from Dutch to English using DeepL (see Methods section in the manuscript for further details).

**Research question**	**Explanation**
1. could the current BCB courses be certified so that these students receive a worthy diploma?	
2. an MBO4 diploma in OST has been given less value. it would be nice if here can be a flow-through to HBO.	
3D printing possible and effective?	
3D scanning	
Develop 3D scanning techniques based on loaded extremities	Loaded 3D scanning is limited in loaded condition of the extremities The better the scan, the better the orthotic design.
3D technology scanning milling and printing	By standardizing processes and products, the focus would increasingly need to be on specific subdivisions of the shoe customization. Whereby time-consuming aspects such as lasts could be omitted
-allergy, regarding material/leather choice	
Create comprehensive matrices that dynamically map confection shoes with a focus on fit	
Alternatives to leather for manufacture shoes	Due to religious or vegan motives of a growing group of users
Burdened or unburdened measurement.	
With foot lever paresis, the question is often how strong or sturdy does the facility need to be to lift the foot lever paresis?	Is it possible to establish a relationship between the rigidity of the facility and the degree of loss of strength?
With many is footwear, there is no real gait linea anymore. While technically the foot can often still do this. Is there a reason for this?	
Many of the patients who are completely dependent on hus OSA also have the need for a kind of HOME SHOES or HOME PANTOFFEL, by wearing these they do not have to wear their OSA until they get into bed, it is technically possible to produce this footwear reasonably inexpensively and deliver it responsibly, yet this is not a standard at many HMO and we therefore have to disappoint patients here!Question: can it be investigated for how much and with what conditions such HOME shoes can be made so that this can become a standard at all HMO in the interest of customers?	REGULARLY this question is asked by customers! Any orthopedic shoemaker will agree, there is clearly a general need especially now that customers also have to do longer and longer with hus OSA.
customer compliance and evaluation -follow-up	A good shoe is only a good therapy if it meets the client's needs and is worn with pleasure and is a real AID to move more comfortably
Conservative versus digital, which is more advantageous?	Many companies find it difficult to move to digital because they think it will cost more. How

	best to convince those companies that digital works better for the future.
That an experienced orthopedic shoemaker be retained for the company to exchange experience with a younger aspiring orthopedic shoemaker.	
That an orthopedic shoemaker be given the space and quiet to measure a pair of shoes on the client.	
The influence of OSA in people with spasm, holding the foot in dorsal flexion at an angle smaller than 90' and its effect. Rehabilitation doctors now often do not support this concept while we achieve good results with it.	
It has been determined by health insurance companies that NO more OPEN orthopedic shoes can be made! Many patients who are completely dependent on osa like Diabetic and vascular problems urgently need to be able to receive an airier osa so that during hot days in NL (and there are more and more of them!) they will experience less sultry feet, this is technically entirely possible > Question: can it be investigated what types of "open" osa can be made and that this is justified for a clearer policy towards the health insurance companies	Many patients who are completely dependent on OSA ask every time if they can also order a breezy osa for the hot days? unfortunately we then have to say no under pressure from the ZV
wearing comfort versus function	
Wearability, comfort of orthopedic shoes.	
Pressure measurements often give good pressure relief results, but wounds still occur. What is the reason for this?	
After amputation of the hallux / TMT 1, we often see that the remaining toes (dig 2-5) show an increase in claw position in the short or long term, causing increased pressure on the PIP joints and the apex of the digits. To prevent that, there could be e.g. Jones surgery of the little toes or by moving the tendon with the so-called Girdlestone-Taylor surgery.The question is, has this ever been researched? If not, could that be a study? If so, does the research group have any data on this that would recommend/disapprove?	
Develop simple efficient low-cost (pressure) measurement methods	
Effect in gait analysis after shoe modifications such as heel raises, unwinding, ankle arthrodesis and so on.	

Effectiveness of orthopedic shoes based on the demand for help, self-defined by the patient and already realistically assessed in the shared desision.	Our measured effectiveness of orthopedic facilities is often much higher than those from studies. Where are these differences? Are the right questions being asked? What is the researcher looking for?
Effectiveness study of wearing orthopedic footwear through data. (Sensors in footwear)	Gain more insight into theory behind shoes
Emperical support for the risk reduction of amputations in a diabetic foot with the timely application of a well-designed orthopedic shoe	
There are already socks in development with temperature sensors all around. I look forward to pressure measuring socks, so not only being able to measure plantar	
functionality cost/benefit	by the insurer looking only at the costs and not the social benefits
Is the trend toward protocolization going to cause a chronic gap between the protocols used and state of the art knowledge based on best evidence.	
Does a reduction in peak pressure also give a reduction in callus formation?	Meanwhile, is it possible to measure friction and pressure? The two components that cause calluses
Like to see nationwide comparison between indication setting and care provided?	To achieve a possible more transparent and simple reimbursement structure, it is nice to know what care is provided for a given indication.
Growth trends: strong increase in children/adolescents in length growth and narrowing of the feet i.c.w. increase in height. How is this taken into account in the OST workshops ?	Prevention protocol ? Biomechanical examination for long-term effects of increase in foot length ? Other loading symptoms at crural fork, calcaneus, midfoot, toes e.g. ?
heel height/heel lift in shoes, why and what is the added value?	
Do orthopedic shoes have as much effect at home as they do in the lab? For example, do certain activities or surfaces affect them?	
Do trial shoes have a beneficial impact on the course of rehabilitation?	In a rehabilitation institution, trial shoes are requested, however, they are not reimbursed by the health insurance company. The reimbursement received for orthopedic shoes does not cover the making of the trial shoes.
Does a cover layer over double padding(ppt and plastazote) have a negative/positive effect on pressure distribution?	
The very best would be to create an algorithm where disorders based on a medical condition that translate into limitations in standing and walking lead to components in shoe prescription.	There is now an incredible amount of knowledge among both orthopedic technologists and physicians, but it is implicit. if it can be made explicit, this will have many advantages for knowledge transfer and preservation, and hopefully there will be more uniformity in shoe

	prescription. This is somewhat analogous to the orthotic handbook also developed in Amsterdam.
The effect of a (distal) settlement on hip mobility in elderly people who sit more than walk with extension limitation in the hip joint	Is it clear to professionals how a settlement affects the entire chain of joints? Would be good to have more insight into this
The use of MCO versus OSA, what do the statistics show?	Is there a contraction of OSA numbers due to the growing market of MCO?
The difference in wearing orthotics in good sturdy shoes and bad not sturdy shoes	Many people have orthotics, but walk on bad shoes
How do people experience custom-made slippers compared to off-the-shelf slippers (better support, comfort, weight)?	
How great is the pressure drop in an orthopedic shoe in different applications of achoena fitting	For example, by using an unwinding bar or soft materials. What effect does it have?
How is the "user environment" an integral part of the information phase in the measurement process?	
How is it to increase satisfaction when using OS?	
How can the acceptance of the device (orthopedic shoe)be improved for people with mild impairment.	I often find that when people have such a severe foot abnormality and depend on the shoes, acceptance is easier than with clients with a slight abnormality and they need this aid to prevent more severe.
how to measure the effectiveness of orthopedic footwear	
how to create acceptance for wearing orthopedic shoes among people who only wear fancy narrow shoes because it suits their dress style.	especially older posh ladies and non-western ladies and gentlemen suffer from this. they don't want shoes because it doesn't match the clothes and their appearance. while there is a real indication that they will walk better and safer, and there is less risk of wounds and amputations if they start wearing orthopedic shoes.
How can it be ensured that people who have little money but are not known to the municipality can still get shoes and not say no because of the money.	this seems to be an increasing problem, on average 2-3 times a week someone reports I can't pay for a replacement pair, or I can't pay for it so I keep walking, with all the consequences. feet being broken etc.
how to create intrinsic motivation to wear orthopedic shoes even in the home in diabetic patients.	
How can the shoemaker's fitting process be more standardized, (perhaps more digitized?), so that faster, more precise with fewer errors (to a pressure adequate) shoe fitting can be arrived at.	

How is it that for some with an osteoarthritis complaint with pain with movement, a stiffening is actually counterproductive.	We regularly remove sole stiffening from a shoe, where the theory says it would be necessary.
How can innovation contribute to cheaper customization?	
How can proper heel lift be objectively determined in people rehabilitating after CVA?	A trade-off must be made between corrected foot position or functional foot position. Now this trade-off is made mainly by the shoemaker based on feeling.
How to get Health Insurer to actually reimburse for the product or service	Health insurers always want a scientific justification for a new product or service.But even if this product, in this case a house shoe meets thorough scientific research, and with a particularly good result it is not reimbursed. The key question, as far as I'm concerned, is what's the point?
How can you make sure you listen more and see where the customer is coming from instead of immediately looking at the technical aspect?	Psychological piece is often forgotten but can offer a lot in therapy fidelity and accepting the change e.g. in cosmetics or being able to change shoes. Emotionally it can be and there is a lot to be gained in the fitting process.
How can you leverage knowledge from others but still differentiate yourself from your competition?	There's a lot of cocky behavior now, and why are you doing it this way because I've been doing it this way for years. All trying to reinvent the wheel instead of using each other's knowledge.
How can we make our field interesting to the new crop of peers?	There are fewer and fewer skilled apprentices, making it difficult to obtain suitable staff.
How to prevent friction/pressure wounds in new orthopedic footwear especially so non-plantar	
How to deal with comorbidities/side pathologies in high-risk DM patients such as acute deformations due to osteoarthritis, edema in high-risk DM patients i.c.w. pressure spots while patients are not yet eligible for new orthopedic footwear/fittings or have just recently received them or that the production time is too long?	Sometimes acute shape changes occur in the feet of high-risk DM patients due to comorbidities or secondary pathologies such as osteoarthritis/RA/edema. In some cases, this can be so acute and drastic that the foot shape changes so much that new orthopedic footwear is needed in the short term. This is to prevent ulcers/pressure spots. The production time of these devices/shoes is often too long, causing the patient to develop ulcers.
How to improve therapy fidelity in Diabetes mellitus patients.	Many DM patients end up not wearing the orth. footwear anyway.
How often does dorsiflexion restriction in BSG pass people who are going to have OSA and what is being done in the field with this?	al podoth. i see many OSA shoes with compensation DF restriction in BSG even when it mi not necessary. This makes the shoes box large and heavy which affects compliance
How far should you go in objectifying the demand for care?	Further objectification generally leads to more measurements. However, taking measurements takes time. If this is overshot, it will not outweigh the benefits: more effective solutions.

How do we improve adherance?	Adherence seems very bad for many people with OS.Acceptance problem?Knowledge problem? Education only works temporarily.
How do costs and quality of life (positive health) compare between higher and lower class OVAC, OSB and OSA (low t/extra High)?	
How do we ensure the quality of our materials when the time the client has to do with the shoes is increasing?	We find that in the marketplace quality is declining
How warm are orthopedic shoes in summer.	many customers complain about high shoes being so hot in the summer
In practice, how are prescribers and shoemakers dealing with efficiency, which needs to be monitored in the context of controlling healthcare costs. Is this reflected in the NVOS foot-shoe protocol?	
What will our market look like in 10 years ;-)Impossible question but: Like to have a broad discussion with insurers on the one hand regarding funding. What can we expect and what is then still reasonably available (price-quality) or other structure. On the other hand, the user, what does he expect from the technologist?	
How much time does a customer really wear their shoes.	Many people have no shoes on in the house, now with corona and sitting at home a lot so few shoes on as well
How much increases fall risk in artificial accelerated or polyphase unwinding.	Often an artificially accelerated settlement is suggested by doctors, but what consequences does this have in terms of falling clients. this is because you see in many especially older people that they are not very stable standing and walking. for the calves a freshened settlement may be better, but is it also wise?
How much reduction in pressure certain elements give at certain foot deformities/peak pressures	
I don't know what the status of the indication matrix is and if any adjustments have been made to it....but on according to me right now a shoe always comes out that falls under the entitlement. According to me there should be stepped care in it	
I would like to research how much influence aesthetics/cosmetics have on the effectiveness of orthopedic shoes.	are shoes better worn as a result? can this be determined numerically? what does it do to the "self-worth" of the client?
In the case of diabetic feet: what is the most suitable material for the shaft? Consider leather, synthetic material,.....	

In how many cases (%) is the footwear protocol fully completed and leads to the selection of the correct product	
To what extent is the orthopedic shoe technician aware of the sims and associated care profile per DM risk patient and the corresponding foot care provided (the treatment plan) which is managed by the (diabetic) podiatrist in collaboration with the medical pedicure/pedicure DM foot.	The orthopedic shoe technician can play a major role in motivating the DM risk pt. to follow these treatment plans as well as possible to prevent diabetic foot ulcers. He can also intervene if he notices stagnation due to circumstances and discuss this with the podiatrist/medical pedicurist. of the
To what extent is a digital fitting system for making lasts pure?	Can you measure a Charcot foot or do an unloaded dimensional measurement and also improves the quality of your finished product and production process time.
To what extent is there sufficient support within the branch / is it realistic to indicate all together in a uniform way, i.e. using the Indication Portal. With the higher goal of becoming better together (at individual level, at company level and at branch level).	I wonder if a substantial portion of companies are willing to invest in this process and share data on the effectiveness of "their" solutions.
To what extent can the digiti be laid pressure-free in pain due to hyperpression?	Often I see problems at the digiti due to hyperpression in people who carry OSAs and people don't seem to be heard in their pain complaints. Are there solutions to this?
How much do the results differ, when doing a measurement with an insole pressure measuring system(e.g.Pedar) and the RS-Scan pressure measuring plate, in production process and final product?	So the difference between these two types of pressure measurement systems in outcome. Does one system provide better and clear information which means, for example, that fewer adjustments have to be made or that certain problems are recognized earlier etc?
To what extent does the orthopedic shoe technician actively follow up with his DM risk patient in terms of follow-up when top layer padding has been used to prevent pressure sores?	Indeed, the diaphos study showed that these polster layers should be replaced/renewed periodically to prevent recurrent ulcers
To what extent are the outcomes of the Pedar syseem useful for rheumatic feet and foot deformities due to trauma?	
Inventory of modular footwear and then the development of a "modular" system that could provide for many more people with foot problems.	OS-a is now too often prescribed. It should be possible to do this much less. There are several "modular" systems on the market, but none are optimal.
influence of the radius of a unwinding device on pressure mtp region and hallux region planetary, and what are the influences on ground reaction forces.	A.d.h.v. pressure measurements we are starting to see a difference influence of shoe adjustments in DM patients with ulcers planetary mtp region and hallux. further research is useful I think. the ground reaction force affects the gait pattern and is measurable, what influences can we capture?
is 3 d scanning as good as gip print to be used for an orthopedic last	Surely the possibilities are more limited with 3 d scanning "what gives the best mold" ?

Is the combination of podiatry and orthopedics an added value over the orthopedic shoe only made from the orthopedic point of view?	In the orthopedic shoe, a podiatric sole can be well incorporated. By bringing the good of these two worlds together, I expect more effect and a more satisfied client.
Is the choice of an orthopedic shoe justified or would a client have achieved the same effect with a semi-orthopedic shoe?	
Is the orthopedic shoes that were fitted necessary or was the problem also solvable with a good sole in a fitting shoe?	
Is the position of the knee joint correctable with a lateral wedge in a person with gonarthrosis.	A simple solution is to use a lateral wedge for gonarthrosis. Only what does this do purely on the knee joint in individuals. Does it actually create a space at the condyles. Isn't an OSA with socket that maintains the position at the ankle better?
Is there already an OSB house shoe? Could that mean the same thing as an OSA house shoe for people with OSBs? (Higher adherence and same pressure profiles as outdoor shoes)	There are OSA house shoes, but this seems useful for people with OSB as well (if it's not already there)
Is there evidence that multidisciplinary care for a client/patient leads to better orthopedic shoes and how is the improvement defined.	Is there a description of a protocol on multidisciplinary approach and choices made in the process followed in determining and designing orthopedic shoes.Are the roles described incl. those of the user?
Is there any possibility of investigating whether orthopedic shoes can be made lighter in weight. This is something people often ask for.	research into lighter running soles that are still wear-resistant.
Is there a standard to be established between the severity of the condition and the necessary solution.	You often see expensive solutions while then my understanding it should also be easier to solve.
Is there a difference in balance during standing and walking between a round settlement and a two-stage settlement?	In particular, I am thinking of a research group comparing people with sensory impairments with people without sensory impairments. I am also interested in the use of ankle strategies versus hip strategies.
Is there a difference in stable balance and in kinematics of the knee and in the 1st ankle rocker in loading response between a haf rounding and a buffer heel with different curves and hardnesses?	
Does the Human and Engineering Orthopedic Shoe Technology program have a curriculum set up regarding the high-risk diabetic foot regarding sims and care profiles?	In practice, trainees/recent graduate orthopedic shoe technologists have very little knowledge of this. While this is essential in the prevention of diabetic foot ulcers.
Is it possible to print a footbed that self-regulates by pressure, i.e. that collapses in places if the pressure in an old ulcer region exceeds 10 newtons?	

Is it possible to make a 3 d impression or scan that corrects or supports any positional deviation in the foot and at the same time performs a pressure measurement to show both the maximum correction and the most optimal pressure distribution and correct leg position during full foot loading. This impression or scan is then the most optimal basis on which to design an individual last.	To remove all uncertainties involved in making a 3D print or 3D scan. To be able to objectify the choices made during sizing and casting and/or scanning to all parties involved To make knowledge and skills needed in the current conventional ways of making 3D impressions accessible to novice sizing technicians To obtain the most optimal combination of correction, support or acceptance of the problematic foot position with the correct pressure distribution in complex foot problems. To allow the client/patient to experience what the future orthotic or shoe fitting can do and thus create greater involvement. To be able to record the measurement data for future checks for aid effectiveness
Is it possible to make and unwinding with choices. Home position unwinding without stiffening outdoor for longer distances with sole stiffening with more unwinding and more rounding. this should be and smarte solution that the customer does not have to adjust himself , the choice makes and algorithm based on the walking pace	
Is it possible to develop good open orthopedic summer shoes that are reimbursed by health insurance companies?	Many patients find closed shoes in summer too hot and very uncomfortable. Health insurance companies do not allow sandals. Is it possible to develop a type of orthopedic shoes that meet quality requirements in all respects while being airy and cool ? Goal: improve compliance wearing OSA in summer
Is it possible to measure shear forces between the foot and the shoe during walking and does this measurement data add value to improve shoe intervention?	
Can the pressure of the shoe on the dorsal side of the foot be measured.	Pressure spots on the dorsal side of the foot.
Can the Stepped Care be worked out for confection, adjustments to confection, OSB, OSA, OSA High and OSA Extra High?For what indications, what solution.	
Can lighter material use equally generate the desired outcome? So that the shoe becomes lighter in weight.	Many people refuse to wear their orthopedic shoes (in part) because they feel too heavy, this applies both indoors and outdoors.
Can't OSB/low complex footwear be used more often where OSA/high complex footwear is now used. In other words, isn't OSA/HCS being prescribed too quickly and easily.	I regularly see people who are fitted with OSA/ HCS, who could very well do with OSB/LCS

Can the response options to care question items in the Indication Portal be further objectified?	In the development of the Indication Portal, the aim is to objectify the demand for care as much as possible so that more direction can be given to the indications resulting from it and that data analysis for the purpose of improvement processes is more effective.
Can OSAV ? orthopedic safety shoes be produced Lighter in weight within NL?	For OSAV ort shoes safety we are 100% dependent on 1 company and they only make very heavy shoes, CAN'T that be made lighter? > yes I think so! Can that be researched
Qualitative study of patients' needs, behaviors and choices regarding what they want and can walk on indoors.	A large group of patients in Amsterdam are used to leaving shoes at the doormat and walking on slippers, socks or bare feet indoors. Health insurance does not cover indoor footwear. The research should identify the needs, desires, expectations and behaviors of different patient groups. It should also identify what patients think they need and how much they would be willing to pay for it.
Lateral and medial lips on orthotics, how effective are they	
Does an orthopedic shoe with adequate prescription lead to better balance during standing and walking in patients with pareses of the lower legs?	
regarding efficacy; broadening of the DIAFOS study; patient characterization (only patients with completed ulcer)	
Regarding effectiveness, broaden DIAFOS research: equipment to be used (is now limited to costly and labor-intensive Pedar system).	We believe that with less costly and flexible, accessible systems, a larger group of users will be reached.
Regarding efficacy, broaden DIAFOS study: patient characterization (is now focused only on patient with relapsed ulcer.	We believe that scientific evidence is so far lacking for a very large group. For this group, proven effectiveness is important for continuity of their care and prevention of deterioration.
regarding effectiveness; broadening of DIAFOS research; characterization of equipment to be used (is now limited to costly e labor-intensive Pedar system)	
Do orthopedic shoes make my foot/lower leg muscles weaker ?	Like wearing glasses often which makes your eyes get used to them,I can imagine that wearing a foot device can make your muscle corset weaker/if so is this not a bad thing ?
MDR and intended use: for all medical devices, including orthopedic footwear with or without CE marking, intended use must be established and documented at the patient level. In addition, clinical evaluation must be available for each medical device.	

MDR and intended use; for all medical devices, including orthopedic footwear with or without CE marking, intended use must be established and documented at the patient level. In addition, clinical evaluation must be available for each medical device.	
More material research regarding the diabetic foot supplement.	
Measurability of shear forces in the shoe.	
With what minimal (shoe) provision (should not be too expensive) is a vulnerable foot sufficiently adequately protected?	
Shouldn't the fitting of an orthopedic shoe in diabetes be accompanied by mandatory advice from a dietitian?	
Possibly not a question for scientific research, but relevant: reassessment of role division of referrer/supplier. Who fulfills the role of referrer (2nd line specialist/podotherapist/shoe technologist) in the future so that this contributes to sensible, adequate and efficient foot care	
Possibly not a question for scientific research, but still relevant: reassessment of referrer/supplier roles. Who fulfills the role of referrer when (2nd line specialist/podotherapist/schootechnologist) and in what way does this contribute to keeping foot care affordable	
Possibility of maintaining bet vocational education (lbo-mbo) in the Netherlands	Increasing dependence on foreign manufacturing industry
Multi functional and multi purpose all-round "sock" giving offloading.	
New revenue model for orthopedic shoe technology.	Traditionally, clients have automatically come to the orthopedic shoe technician. I expect that the health insurance companies will reimburse less and less and that people will have to pay more and more themselves. Hence research question 2. The management should be more focused on bringing in clients, a.o. through total foot care. See the management in Australia. The websites alone clearly show the differences.
New materials and production methods.	Shoe orthopedics is still very old-fashioned. Shoes should be able to be much lighter. Manufacturing methods need to be improved; 3D printing?
Whether a donkey ear to the supplement is more effective than when incorporated into the shoe.	
off the shelf shoes/semi-finished products possible?	

In order to make orthopedic shoes, good materials must be available at an orthopedic shoe repair shop to make a pair of good shoes.	Bv Materials NeededGood and strong materials (leathers) and the like present, available to eventually make the shoes.
Research on new self-adaptive materials in orthopedic shoes	
Develop a useful method for measuring and being able to improve the unwind correction of footwear.	Good smooth unwind correction is important among other things in forefoot problems and when using a sole suspension. Many OSA and OSB have moderate to poor settlement, often starting too early and slowing down soon after. It is desirable that a workable method be developed that will make it easier for shoemakers to provide footwear with a good smooth unwinding correction.
Develop a simple communication tool for prospective shoemaker clients that answers all kinds of questions such as co-payments, what a person is entitled to, what one can or cannot expect from shoes.	Many customers in Amsterdam appear to have wrong expectations about orthopedic shoes. People are mainly concerned about the appearance of the shoes. Insufficient is known about what a shoe can do and what it will cost.
Development of a new work system in the Netherlands with the goal: total foot care. All professionals around foot problems come together in this. This starts with the development of a gatekeeper.	Foot care is fragmented and there is little mutual cooperation. This does not benefit the patient
Based on what criteria is the prescriber making the indication OS (OVAC, OSB, OSB modular concept, OSA)?	
At what point is it the best thought to switch from a sturdy walking shoe with orthotics to orthopedic footwear	
In what way is lifestyle or daily activity pattern of the user taken into account in the technical design and production of the orthopedic shoe.	Is it taken into account whether someone walks a lot or a little,is active or not, and therefore there are different user requirements when making the shoes. Is there a user profile or any data on that?
Patient empowerment in relation to collaborative decision making: what does one need to have a say in their care and resources?	
Patient empowerment in relation to collaborative decision making; what does the client or patient need to have a say in their care and resources?	
-Polster materials supplements application, elaboration pressure distribution.	
product development 3D printing technology	Particularly in material development for JetFusion printing technology
project pressure measurement during fitting. is it possible to reduce the pressure in the later shoe with a set-up with an integrated	

pressure measurement on which cast or scanning is done?	
Realize insurer-reimbursed form of adequately OSB house shoes	Steps have been made for individual home shoes, these are not reimbursed. Also for less abnormal but vulnerable feet, shoe facilities must be made available, what requirements must these meet and how can we realize that facilities can still be provided.
Working together structurally with podiatrist I would love, protocols, ideas work instructions a foot portal to work together...Is there a need for that?	to make the client move better, I think both our vision is complementary to move forward together with the client one step further
Sensors in shoes in high-risk patients, identifying "hazards" and making prognoses.	
Standardization of different facilities (solution approaches) in different pathologies. Insightful for the chain.	There is a lot of "tinkering" in the industry that results in different solution approaches and views. This does not always benefit the patient. The new indication protocol is already a nice step forward. Now for the next step in protocolization.
Do the current insurance terms for new orthopedic footwear/provisions for DM high-risk patients care profiles 3 and 4 match the "shelf life" of orthopedic footwear and its supplements? Or do these high-risk patients require a modified delivery period of to better prevent recurrence of ulcers?	Foot clinics and practices often see recurrent ulcers developing in existing footwear that has reached the end of its useful life, while new orthopedic footwear is already in production or while the patient is not yet entitled to new shoes/provisions. For the podiatry/foot clinics, it is then often a case of "biding your time" until the new footwear is ready.
To measure shear between foot and orthosis	
Accessibility orthopedic shoes earlier in the chain: what is the effect t.a. Application stepped careb. Degeneration of complaintsc. Recurrence of complaints.	
Access to orthopedic shoes earlier in the chain; what is the effect regarding 1) application of stepped care 2) degeneration of complaints 3) recurrence of complaints	
top 5 digital in shoe pressure measurement systems. Identifying all pros and cons. Monitor user experiences	
Many patients experience orthopedic footwear as heavy and others do not. What is the reason for that?	Does it depend on the type of footwear they are used to. With certain conditions more?
Many shoes are fitted/or provided with a standard heel height of about 1.5 cm. What is the effect of heel height on forefoot loading?	Patient provision of shoes with different heel heights in the last and pressures assess.
mostly it seems as if prosecuting an OS is not done, there seems to be no established quality system with monitoring of the	experience....often I pick up the phone to get people to evaluate with the OS maker in question

customer and adjusting and or intervening where necessary to have compliance high	
Often orthopedic footwear is perceived as heavy. How can production techniques used in the sports shoe industry be applied in our current industry?	See research question
Comparative study between podiatric and the soles fitted and made by an orthopedic shoemaker on effectiveness (Qualitative study on reduction of symptoms?) and offloading abilities.	Insurances nowadays often reimburse from the basic insurance through the podohterapeut orthotics and then not orthopedic shoemaker soles.While the effectiveness of both is still insufficiently tested and never compared.The quality is very variable, the offloading qualities are also not tested.Podo soles are worn in ready-to-wear shoes, orthopedic shoemaker soles too. What are the differences?
Question is how is it possible that podiatrists at at least two Health Insurers, are allowed to prescribe orthopedic shoes without being trained to do so?	A podiatrist lacks sufficient knowledge to write a shoe prescription/treatment plan
Why is it that insurance companies, are increasingly pushing shoe reimbursements down, while OSA/SOS is only a drop in the bucket in health costs in percentage terms?	
As an insured person, why does my health insurance company only allow me to have 1 pair of orthopedic shoes fitted every year and a half?	In fact, this is not conducive to the shoes if you have to wait a long time for the end result that a pair of shoes were fitted for me as an insured. This often takes more than two years. The previously fitted pair of shoes are then often subject to wear and tear.
Why orthopedic footwear are not worn in practice and what can be done about it.	
Why are we as orth shoe makers and companies bombarded every year by the health insurance companies that the cost price of the shoes has to be lowered further, while it has become clear that the quality of the product and the aftercare for the patient goes down as a result? this has led in recent years to a great STRESS among the companies and professional staff that is already very difficult to find today. the increasing pressure to work with less time and inferior materials, customers have to do longer and longer with their shoes with the risk of foot problems and less mobility. when will this stop and can there be a mutual RESPECT between both parties, it now seems like a price war over the heads of the patients! Question! Can there be an independent investigation into a reasonable price for the various types of orthopedic shoes	Many smaller companies have already been denied contracts by the big insurance companies in recent years if they would not drastically lower their prices that the ZV determines and because of this have been forced to quit, only the big shoe companies can survive this way...and even they have GREAT difficulty in finding a balance between QUALITY and RENDERING. example: LOW OSA reimbursed by VGZ is almost at the COST PRICE! so a little profit very nil and no room for important NAZORG! The latter does belong to a good COST PRICE calculation.

as a kind of guideline for the future and thus unambiguous price agreements instead of the current MACHINES display of health insurance?	
Why are Orthopedic shoes type A often so plump	There is very much an attempt at an OVAC,OSB/MCO to make it an invisible adjustment. Then when someone has to go to full Orthopedics it becomes big, solid, bulky.
When a patient has DM and needs OSAV there is no way to adjust these shoes for Diabetic feet, thus has bad luck and greater chance of pressure spots. Question: why can't OSAV be made lighter and ALSO for DM feet? I think that should just be possible!	The fact is that OSAV cannot be adjusted for DM feet and are also always too heavy.
When do you transition from OSB to OSA	
When to intervene with what degree of foot position abnormalities in children when there are no obvious symptoms, or care needs.	During my children's consultations, I often encounter children who do not have a medical problem or complaints, but who do have an anomaly. Do you always have to fit a corrective device to prevent worse or complaints in the future?
When to provide someone with shoes for nerve loss and when to opt for orthotics	Often this is by feel of the physician and the orthopedic technologist present
When or not surgery for erosive RA in the mtp joints . If yes which technique?	
What does automation bring to the future within the industry?	As more and more is outsourced and technology continues to advance, I ask myself what happens to craft.
What does the location of the Apex of the settlement do on the range of motion of the upper ankle joint	
What does an unwinding device in osa do to flexion and extension in the knee	I see settlements coming by where the knee is pushed so much into a certain position that I would like to see more data on this to make it transparent
Which gives the difference between a built-in EVO in the orthopedic shoe compared to an orthopedic shoe with a loose EVO in it.	

What is the (cost) effectiveness of orthopedic footwear interventions in people with diabetes mellitus and foot complications?	
What is the (cost) effectiveness of orthopedic footwear interventions in people with an increased risk of falling?	
What is the effectiveness of footwear solutions based on a corrected plaster cast versus footwear solutions based on a digitally corrected scan of an uncorrected foot?	Either the change taking place within the industry is an improvement (more effective and/or efficient) or there are other motives.
What is the average period of use for OS? Please break down by different diagnosis groups. For example, disability care (Wlz)?	
What is the ideal height of the shaft device in a charcot deformity ? Especially for an initial or repeat provision, and what is the best material for a shaft provision ?	In daily practice, the shaft height of a Charcot device is sometimes a matter of discussion. Does the device have to be so high? Can't it be lower? The client experiences the device as heavy and cumbersome.
What is the added value statistically of the Pedar measurement.	
What is the added value of making a VLOS versus cast treatment in patients with diabetic ulcers?	
What is the added value to the user when using the facility:-Pain score reduced?-Mobility improved?-Wound healing?-Less medication (painkillers/inflammatories)?-Less other care needed?	Would like nationwide monitoring.If values can be linked to this, any added value and/or cost savings of a facility can be demonstrated.
What is the most ideal shoe closure for people with half-sided paralysis, assuming that these people put on their own shoes.	Closure is almost always a problem for people with half-sided paralysis; it would be nice to have a solution for this.
What exactly is needed in osteoarthritis in the OSG with no instability symptoms at all. Is it then already sufficient to have an OSA with artificial slope and firm contrefort sufficient	
What is the average lifespan of each type of shoe, and what factors affect severe excessive wear.	
What is the best time to start an orthopedic shoe after cast treatment for a diabetic ulcer?	
What is the effect on cover supplement. Leather or e.g. poron Rosa with plastezote. I so often see soles that are terribly hard bv shore 60 with a very atrophy vegetable kapiton. With substantial pressure spots.	
What is the effect of wearing orthopedic footwear icm treatment physio/ergo therapy	By linking a "course" dealing with tool to product
What is the effect of a settlement facility?	With respect to pressure under the forefoot, as well as with the ROM of ankle, knee and hip
What is the effect of ankle wraps on the ankle joint	what is the difference in effect with a flexible or rigid ankle closure

What is the effect of different supports in the footbed/sole?	for example, an RCTB/Pelot
What is the difference in off-loading and prevention of shear forces between an enclosure between liner and over-leather or a circular enclosure in amputation of part of the forefoot?	In practice, I often use a circular surrounds (removable socket + tongue) in amputations of part of the forefoot (such as an amputation of TMT 1.)from the idea that it absorbs shear forces better than if only a supplement is used or a socket between lining and over leather. i would like to see scientifically proven which method produces the best results in preventing shear forces and which contributes to optimal pressure distribution.
What are the expectations?	
What else can be done in patients with sweaty feet to reduce this.	Sweaty feet can cause softening.
What can you expect from repairs and modifications to the OS?	
So what makes my shoe a quality product?	So many contractors so many opinions on what a shoring should look like. I would like to see more measurable whether a solution is actually a good product. For insurance but also for patient to be able to be faithful to therapy, if you know that that real looking orthopedic shoe is really going to help you because this has been proven by research.
How does the client feel about longevity?	
What shore value is needed to make an orthotic for someone with diabetis	
What are proven effective interventions to increase compliance for using optimal offloading (orthopedic shoes)?	
What are the ideal construction materials for a sports inlay ?	
What are the short-, medium- and long-term effects of 3D printed scaffolds that serve as soles under a diabetic shoe ?	Shore values, density of material, elastic properties etc. determine the frictions around the foot under load and movement. What gives the least disruption to tissues around the foot ?
What are the minimum requirements for a house shoe/home slipper in patients with diabetic mellitus and polyneuropathy.	Users indicate that they do not wear the shoes in the house, as they find them too stiff, cannot get in and out quickly and prefer felt materials.
What are the differences in the effectiveness of different implementations of ankle enclosures, in terms of the combination of material use and design?	I think it would be interesting to know what forms of implementation are used within the industry and what the differences are in effectiveness.
we have many shoe solutions but several are not substantiated and are chosen/advised on experience.would like to see more substantiation.	
wearables with graphene as a basis for making a corrected print	

What material and thickness in artificial running gives most or least pressure reduction under the forefoot	
What material choice in footbed of OSA give most reduction of pes cavus foot type with a plantar fascia	
What material research under the OSA is light in weight and still gives sufficient foot lift in light foot lift failure and diabetes.	In diabetics, for example, due to neuropathy and loss of strength, you want to choose an OSA with a rigid sole and possibly built-in evo for foot lift. But often due to weight the patient switches to a less adequate alternative.
Which type of shoe is most commonly prescribed nationally and statistically for which medical indication(s).	
Which 3D scanner is best to use as a fitting technique?	There are many different systems on the market, how do I know I am using the right one and it is not harmful to my patient, e.g. the laser light.
Which fitting method/ product gives the best lasts?And RESULTS in best supplements with OSA	
What biomechanical settlement in various dm ulcers	Much difference in vision per shoemaker
What hardness of undersole works best for dm patients	
What materials can make the orthopedic shoe device lighter in weight to increase compliance	
What normalization is possible for the ratio of (peroneal) provision height tav foot length in flaccid paresis?	The height of a peroneal nerve (provision) is often chosen for different reasons. some look at the height of the thickest part of the calf so as not to end up around the Achilles tendon, and others look for the limit because the customer wants the lowest possible shoe. a loose provision in s the shoe will soon be higher than one that is processed in the shoe. but what is the minimum height that the provision in the shoe should have without getting defects to the skin, etc.?
What practically applicable classifications are there to classify foot shape abnormalities in orthopedic shoe practice?	
Which type of settlement relieves the forefoot the most?And does everyone rub it the same?	
What outcome measures are appropriate to assess the effectiveness of orthopedic shoe therapy interventions and how can they be applied in practice?	The indication portal would like to make data-driven improvement suggestions for 'best practice,' this requires uniform outcome measures and and a uniform measurement method
scientific research on use of food (veganism) and the subcutaneous tissue under the feet	in my opinion, the subcutaneous tissue migrates and is thinner

Scientific research on the use of asthmatic products and heel pain complaints/ plantar fasciitus	
Scientific rationale for various (commonly used) orthopedic devices in a predetermined condition.	The products used in orthopedic shoe technology are mostly not validated. It would help tremendously if the effect of orthopedic devices were demonstrated.
Are there data on daily use and feedback from users of orthopedic shoes; user loyalty, user experience, satisfaction and effectiveness?	How is feedback from a user monitored and used to improve the shoes they wear. Not only at delivery, but also in continued use and at visits to the clinic.
can gradations or degrees of severity be indicated when a particular functional limitation gives rise to a particular orthopedic (partial) solution?	The confection is getting better and better, and semi-orthopedic is often confection in execution as well. In order to keep care affordable, you particularly want to pay for necessary individual solutions and not for care that is increasingly common, accessible and available.
Besides the Pedar Novel insole pressure measuring system, are there other insole pressure measuring systems that are qualified to be/have in use?	This is in response to the requirement from the VGZ. Why specifically Pedar and isn't there another system that works just as well but is simpler and cheaper (in the interest of the smaller orthopedic companies)?
Are there guidelines for an adjustment to be performed, adhv a obtained insole pressure measurement?	
Are there differences between pressure relief, progression of plantar pressure, progression of settlement when using a wedge heel compared to a block heel.M.e. also in people with ankle pain	question stems from empiricism (and own experience as a user):people who are used to a wedge heel and once choose a block heel (appropriate to chosen model) have more hindrance of ankle pain - have more hindrance of midfoot - 'harder touch'. can this be supported with objective data?
Are leather, handcrafted shoes more effective for posture and movement, than convenience shoes (e.g. sneakers). What are the long-term effects with wearing both ?	Many children and adolescents walk on "fashionable and trendy" shoes. See studies on attitude by Piet van Loon. These non-breathable products also cause bacterial growth and mold.
Summer shoes are also important for patients with asensitive feet, (such as diabetic patients). What are the requirements, which allow reimbursement by the health insurance company ?	Sandals are not reimbursed, loose slippers are inadequate, loafers uberhaupt not good.OSB like devices, with small ventilation openings and orthotics that can be adjusted, could be adequate. Find out what the requirements are for this adapted/measured footwear to be reimbursed.
Should orthopedic indoor shoes be added to the palette of orthopedic footwear solutions as an effective solution?	Clients frequently request orthopedic shoes with functional requirements specifically for indoor use. This can increase the intensity of use, thereby increasing prevention, especially in diabetics.
