# Supplementary material for: Development of a research agenda for medical grade footwear in the Netherlands: A multidisciplinary multiphase project to determine the key research questions to advance scientific knowledge in the field
Source: J Foot Ankle Res. 2024 Jul 2;17(3):e12016. doi: 10.1002/jfa2.12016 (PMC11633342; doi:10.1002/jfa2.12016)
Supplement: Supplementary file 2 — Supporting Information S2 [file JFA2-17-e12016-s003.pdf]

Additional File 2 – part of the manuscript "Development of a research agenda for medical grade footwear: a multidisciplinary multiphase project to determine the key research questions to advance scientific knowledge in the field"

Additional file 2: Search strategy and search results literature analysis

The published scientific literature was searched on May 28, 2021. The database PubMed, via Medline, was used for this purpose, with the following search terms:

((systematic [ti] AND review [ti]) or meta-analysis [ti] or meta-analyses [ti]) AND (footwear OR shoes OR 'foot orthoses')

This yielded 215 results. These were screened by one reviewer. Finally, 48 systematic reviews were included (Figure 1).

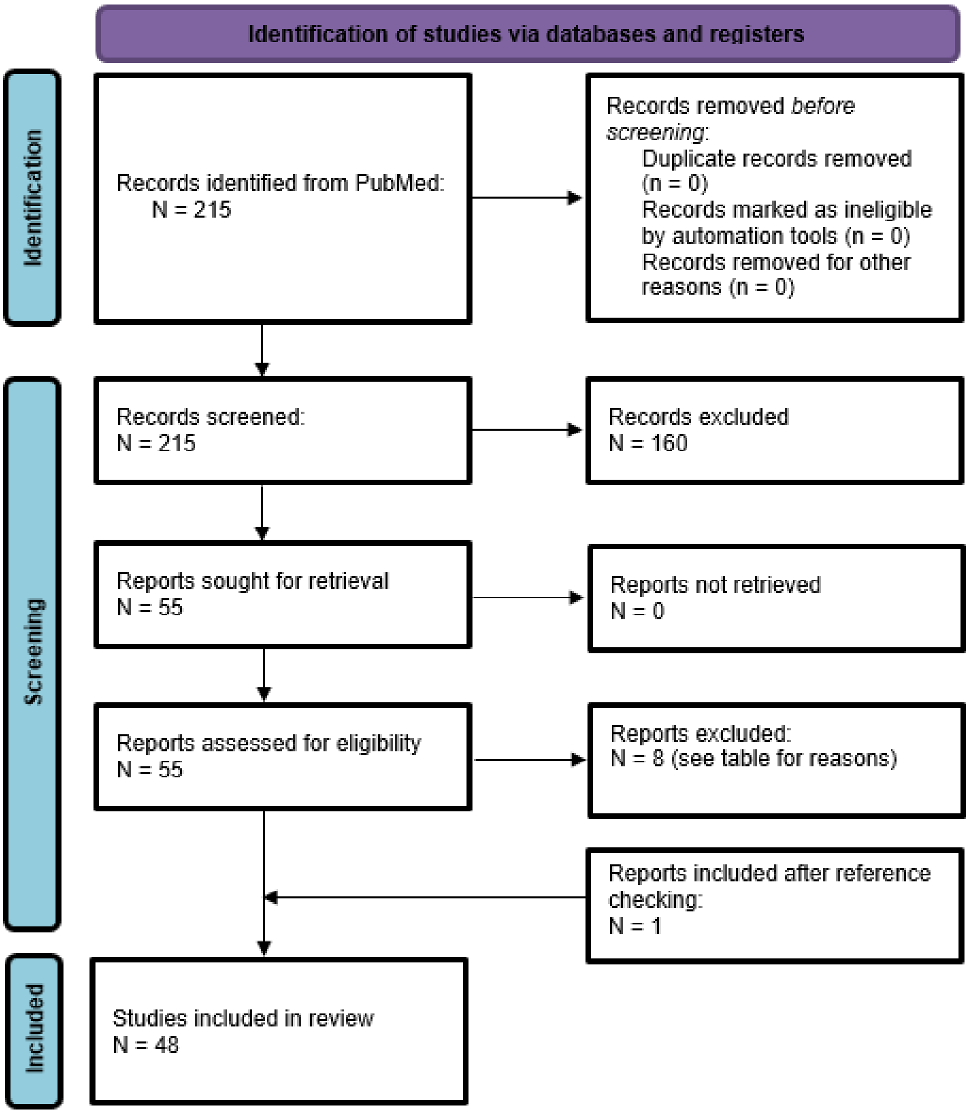

Figure 1: PRISMA flow diagram showing results search strategy

The 8 excluded systematic reviews are listed in Table 1, including rationale. The 48 included systematic reviews, including their findings, are described in the subsequent pages.

Table 1: Excluded papers, with reason

**Paper**	**Ref.**	**Reason for exclusion**
Aboutorabi 2016	(1)	Only studies with healthy subjects included
Bai 2019	(2)	More recent SR available, containing the same and more papers
Bonanno 2017	(3)	Only studies with healthy subjects included
Heuch 2016	(4)	More recent SR available, containing the same and more papers
Healy 2013	(5)	More recent SR available, containing the same and more papers
Ivanyi 2015	(6)	No studies surrounding shoe solutions included
Korada 2020	(7)	More recent SR available, containing the same and more papers
Paton 2011	(8)	More recent SR available, containing the same and more papers

Note: SR = systematic review; Ref = references.

References:

1. Aboutorabi A, Bahramizadeh M, Arazpour M, Fadayevatan R, Farahmand F, Curran S, et al. A systematic review of the effect of foot orthoses and shoe characteristics on balance in healthy older subjects. Prosthet Orthot Int. 2016;40(2):170-81.
2. Bai DY, Yuan ZG, Shao JJ, Zhu T, Zhang HJ. Unstable shoes for the treatment of lower back pain: a meta-analysis of randomized controlled trials. Clin Rehabil. 2019;33(11):1713-21.
3. Bonanno DR, Landorf KB, Munteanu SE, Murley GS, Menz HB. Effectiveness of foot orthoses and shock-absorbing insoles for the prevention of injury: a systematic review and meta-analysis. Br J Sports Med. 2017;51(2):86-96.
4. Heuch L, Streak Gomersall J. Effectiveness of offloading methods in preventing primary diabetic foot ulcers in adults with diabetes: a systematic review. JBI Database System Rev Implement Rep. 2016;14(7):236-65.
5. Healy A, Naemi R, Chockalingam N. The effectiveness of footwear as an intervention to prevent or to reduce biomechanical risk factors associated with diabetic foot ulceration: a systematic review. J Diabetes Complications. 2013;27(4):391-400.
6. Ivanyi B, Schoenmakers M, van Veen N, Maathuis K, Nollet F, Nederhand M. The effects of orthoses, footwear, and walking aids on the walking ability of children and adolescents with spina bifida: A systematic review using International Classification of Functioning, Disability and Health for Children and Youth (ICF-CY) as a reference framework. Prosthet Orthot Int. 2015;39(6):437-43.
7. Korada H, Maiya A, Rao SK, Hande M. Effectiveness of customized insoles on maximum plantar pressure in diabetic foot syndrome: A systematic review. Diabetes Metab Syndr. 2020;14(5):1093-9.
8. Paton J, Bruce G, Jones R, Stenhouse E. Effectiveness of insoles used for the prevention of ulceration in the neuropathic diabetic foot: a systematic review. J Diabetes Complications. 2011;25(1):52-62.

Outcomes literature analysis

The literature analysis searched for "systematic reviews" in the field of medical grade footwear. This means that we specifically looked for articles that provided a systematic review of the existing literature.

The findings are presented in 3 tables. Table 1 shows the findings of systematic reviews of studies done in adult populations, Table 2 shows the findings of systematic reviews of studies done in pediatric populations, and Table 3 shows the findings of systematic reviews on studies investigating methods or materials.

In each table, outcomes are classified by condition (first column). If there was an explicit difference within a condition based on outcomes, or if a systematic review focused explicitly on only one outcome measure, the findings are also classified by outcome (second column). If several systematic reviews focused on the same patient population, and if applicable also on the same outcome measure, it was examined whether there was any difference in the original studies included. If this was not the case, only the most recent systematic review was included (see page 2 for the list of excluded articles). If two or more systematic reviews included different original studies, all were included (third column). When determining the total number of studies per topic, each original study was counted only once (fourth column).

The main findings in the various systematic reviews are described in the fifth column.

Suggestions for future research as made in the discussion/interpretation sections of the systematic reviews are described in the sixth column.

Table 1: Evidence table "review of systematic reviews" - adult populations

Patients	Outcomes	SR (reference)	N[a]	Findings	Future research
**Osteoarthritis**	Kinetics / kinematic, pain	Zafar 2020 (1)<br>Shaw 2018 (2)<br>Healy 2018 (3)<br>Xing 2017 (4)<br>Tan 2016 (5)<br>Arnold 2016 (6)<br>Parkes 2013 (7)<br>Radzimski 2012 (8)<br>Raja 2011 (9)	60	- Lateral wedged insoles may have small beneficial effects on kinetics and kinematics<br>- Evidence of effectiveness of lateral wedged insoles on pain is inconclusive<br>- Masai Barefoot Technology shoes have no effect on step length or walking speed	- Investigate targeted use of insoles in specific biomechanical phenotypes<br>- Higher quality studies are needed<br>- Longer follow-up is needed<br>- Comparator condition is important (trials using neutral insole rather than no insole as comparator show generally no or smaller effects of lateral wedged insoles)
**Diabetes and/or neuropathy**	Ulceration	Ahmed 2020 (10)<br>Alahakoon 2020 (11)<br>Crawford 2020 (12)<br>Van Netten 2020 A (13)	11	- Therapeutic footwear may reduce the risk of ulceration	- Uncertainty remains, due to heterogeneity, differences in usual care and possible publication bias; new trials needed<br>- Combining footwear with adherence-improving interventions
	Pressure	Collings 2021 (14)<br>Ahmed 2020 (10)<br>Van Netten 2020 B (15)	57	- Therapeutic footwear is effective in reducing pressure<br>- Evidence available especially for rocker soles, algorithm-based design, arch profiles, metatarsal additions and insole modifications<br>- Insufficient evidence for effect of casting techniques or specific materials on pressure	- Investigate effect of following parameters on pressure: height of shoe, heel or toe; upper and sole materials; heel counters; closure systems<br>- Personalized approach<br>- Activity-specific designs<br>- Studies using a controlled design remain needed<br>- Systematic approach to combine various features within shoes/insoles is lacking in studies<br>- Develop core outcome set for plantar pressure
	Balance and gait	Horstink 2021 (16)<br>Paton 2016 (17)	15	- Static balance: improves with vibrating insoles, no effect of top cover, reduces with rocker bottom<br>- Gait: limited evidence of small effects on variety of outcomes	- Studies with higher quality designs are needed<br>- Studies outside the laboratory are needed<br>- Comparison of devices with different mode of action (mechanical support vs sensorimotor control)<br>- Investigate economic and clinical effectiveness, and usability and acceptance of devices
	Adherence	Jarl 2016 (18)	6[b]	- Non-use: 27-40%<br>- Patient's perceptions and conditions associated with non-use	- Use a theoretical model<br>- Investigate perceived costs and benefits<br>- Investigate associations of non-use with social and economic circumstances or health-system

**Multiple pathologies**	Adherence	Swinnen 2015 (19)	7[b]	- Non-use: 5-23%<br>- Variety of reasons / associations with non-use	- Standardized adherence assessment<br>- Investigate associations with disease severity, social situation, and time since prescription
**Rheumatoid arthritis**	Pain	Tents-Diepenmaat 2019 (20)<br>Arias-Martin 2018 (21)<br>Gijon 2018 (22)<br>Tents-Diepenmaat 2018 (23)<br>Sena da Conceicao 2015 (24)<br>Hennesey 2012 (25)	19	- Pain reduction following provision of orthopaedic shoes<br>- Weak evidence to support the effectiveness of custom foot orthoses on pain reduction	- Comparing effectiveness of therapeutic footwear with a control condition<br>- Responsiveness of outcome measurements to changes over time is largely unknown<br>- Investigate effect of shoe characteristics on outcomes<br>- Investigate (cost-)effectiveness of stepped care approach
	Pressure	Tents-Diepenmaat 2019 (20)<br>Arias-Martin 2018 (21)<br>Gijon 2018 (22)<br>Tents-Diepenmaat 2018 (23)<br>Sena da Conceicao 2015 (24)<br>Hennesey 2012 (25)	9	- Therapeutic footwear may reduce high pressures<br>- Weak evidence for custom orthoses to decrease forefoot plantar pressure<br>- Soft orthoses may better reduce forefoot plantar pressure compared to semi-rigid orthoses	- Pressure is hardly studied<br>- Comparative effectiveness of insoles and their characteristics is needed<br>- Investigate sophisticated construction methods
	Function	Tents-Diepenmaat 2019 (20)<br>Arias-Martin 2018 (21)<br>Gijon 2018 (22)<br>Tents-Diepenmaat 2018 (23)<br>Sena da Conceicao 2015 (24)<br>Hennesey 2012 (25)	19	- Therapeutic footwear may improve foot function and physical functioning<br>- Inconclusive evidence for custom foot orthoses on function	- Comparing (cost-)effectiveness of therapeutic footwear or foot orthoses with one or multiple different control conditions
	Gait	Arias-Martin 2018 (21)<br>Sena da Conceicao 2015 (24)<br>Hennesey 2012 (25)	8	- Custom orthoses showed inconclusive evidence for gait parameters and walking speed	- Variety of parameters investigated, with limited possibility for comparison
**Plantar fasciitis**	Pain, function, range of motion, plantar pressure	Schuitema 2020 (26)	28	- Mechanical treatment (including insoles and shoe adaptations) seems beneficial in relieving symptoms related to plantar fasciitis<br>- No difference in effect of different insoles (prefabricated vs. custom-made; full length vs. heel cup)<br>- Some evidence for rocker-soled shoes in achieving positive outcomes	- Various methodological limitations (high risk of bias [primarily: lack of blinding, including co-interventions]; no objective outcome measurements) that need to be solved in future research

**Flat foot**	Pain, function, gait	Gomez-Jurado 2020 (27)<br>Desmyttere 2018 (28)<br>Banwell 2014 (29)	24	- Foot orthoses may reduce pain<br>- Foot orthoses improve physical function<br>- Foot orthoses with medial posting may improve kinematics<br>- Foot orthoses do not seem to have an effect on kinetics	- Some studies also involved home-based exercise programs, in combination with orthoses<br>- Higher quality studies needed<br>- Adherence to wearing is important, as well as the footwear in which the orthoses are worn, and this should be better investigated<br>- Insight into effects of design, materials or production is needed<br>- Personalization of orthoses is needed
**Plantar heel pain**	Pain, function	Babatunde 2019 (30)<br>Rasenberg 2018 (31)<br>Whittaker 2018 (32)	22	- Foot orthoses may reduce pain in the medium term (when defined as 7-12 weeks), but not in short- or longer-term<br>- No evidence that foot orthoses improve function<br>- No difference between customized or prefabricated orthoses<br>- Orthoses are not superior nor inferior to other commonly used treatments	- Higher quality trials needed<br>- Orthoses should be part of multimodal treatment approach, also investigating mode of delivery, dosage, timing and intensity<br>- Comparison with 'wait and see' or 'no treatment' as control is lacking for studies on foot orthoses
**Lower back pain**	Pain and function	Kong 2020 (33)<br>Chuter 2014 (34)	16	- Unstable shoes may improve function but not pain, compared to regular shoes<br>- Custom-made orthotics may improve pain and function, compared to no orthotic<br>- Insoles or orthoses do not seem to prevent lower back pain	- No evidence for long-term effects, this should be investigated<br>- Classifying patients into subgroups and prescribing treatment using clinical prediction rules
**Leg length discrepancy**	Pain, function, and gait	Campbell 2018 (35)	10	- Low quality evidence that shoe elevators reduce back, hip and knee pain and improve function in people with lower leg discrepancy	- Conflict about magnitude of leg discrepancy to be considered normative<br>- Inconsistency in proportion of leg discrepancy to correct<br>- Most studies are old; high-quality studies with adequate controls are needed
**Parkinson's**	Balance and gait	Alfuth 2017 (36)	3	- Textured/stimulating insoles do not improve balance<br>- Textured/stimulating insoles do not improve cadence or step length, but they may improve gait velocity	- Higher quality studies needed (longer follow-up, randomized)

**Multiple sclerosis**	Balance and gait	Alfuth 2017 (36)	3	- Textured/stimulating insoles do not improve balance or gait	- Higher quality studies needed (longer follow-up, randomized)
**Hallux valgus**	Skeletal geometry, pain, satisfaction	Hurn 2020 (37)	3	- Foot orthoses do not improve hallux valgus angle<br>- Foot orthoses may reduce pain in the intermediate term, but surgery may reduce pain to a greater extent	- High quality studies needed<br>- Self-report frequently not included as outcome<br>- Interventions might be more effective when targeting a younger population with less severe hallux valgus
**Achilles tendinopathy**	Pain	Scott 2015 (38)	3	- Foot orthoses may not be better than no treatment	- High quality studies needed<br>- Multifaceted treatment approaches should be studied
**Anterior knee pain**		Collins 2012 (39)	2	- Foot orthoses may reduce pain compared to placebo, but physiotherapy is superior in pain reduction	- Higher quality studies needed<br>- Foot orthoses only to be studied as an adjunct treatment, in addition to evidence-based physiotherapy

Note: SR = systematic reviews; [a] : N given is number of included studies in the combined systematic reviews; [b] : one study included in Jarl 2016 was also included in Swinnen 2015.

Table 2: Evidence table "review of systematic reviews" - pediatric populations

Patients	Outcomes	SR (reference)	N[a]	Findings	Future research
**Flat foot**	Skeletal geometry, pain, function, kinetics	Choi 2020 (40)<br>Hill 2020 (41)<br>Dars 2018 (42)	18	- Foot orthoses may have a positive impact across a range of outcomes, but uncertainty remains.<br>- No strong evidence on long-term improvements in outcomes.<br>- Separated on footwear type: no evidence for corrective footwear, some evidence for functional footwear.	- Methodological concerns, higher quality studies needed<br>- Include standardized diagnostic parameters and outcome measures in future studies<br>- Longer follow-up to investigate sustained impact<br>- Only investigate effect of orthoses in children aged 10 or older<br>- Extensive radiographs needed as outcome<br>- Comparator condition of standard footwear always needed
**Cerebral palsy**	Skeletal geometry, kinetics	Hill 2020 (41)	4	- Sagittal stability footwear could tentatively be recommended over standard retail footwear for AFO footwear combination in children with CP.<br>- Instability footwear may improve dynamic balance.	- Multiple methodological concerns, higher quality studies needed<br>- Longer follow-up to investigate sustained impact
**Hypotonia**	Alignment, gross motor function, gait	Hill 2020 (41)<br>Paleg 2018 (43)<br>Weber 2014 (44)	7[b]	- Foot orthoses may modify structural alignment.<br>- Foot orthoses might be beneficial for gross motor development, provided a child can walk independently when the orthosis is prescribed.<br>- Foot orthoses do not improve gait parameters significantly.	- Overall quality poor; higher quality studies needed, both in methodological detail given and in using comparator conditions<br>- Debate on difference in outcomes between supra-malleolar orthoses vs. foot orthoses; currently unclear which may work better<br>- When is the optimal time to introduce orthoses?<br>- How to combine orthoses with physical therapy?
**Juvenile idiopathic arthritis**	Pain, quality of life	Healy 2018 (3)<br>Fellas 2017 (45)	3	- Customized foot orthoses may have a small effect on pain reduction.<br>- Customized foot orthoses may improve foot function.<br>- Customized foot orthoses do not have an effect on quality of life.<br>- Customized foot orthoses may improve walking speed.	- Based on 2 RCTs primarily focused on orthoses, so more studies remain needed<br>- Orthoses compared as part of holistic care in 1 pilot RCT; however, not all participants received orthoses in that trial, and some received an orthoses in usual care, which may obscure effects<br>- No trials where physical therapy was also included as part of holistic lower-extremity/foot care

					- Clinical relevant effect on pain reduction, but statistically not significant, which implies that larger sample sizes may be needed
**Calcaneal apophysitis**	Pain, physical activity	James 2013 (46)	6	- Orthoses provide greater pain relief than heel raises.<br>- Orthoses with a brim (heel cup) and medial arch support was most effective.	- Prefabricated orthoses have not been studied<br>- Potential selection bias in studies with over-representation of children active in competitive sports, research in broader populations needed<br>- Determining foot type and/or activity level that is more receptive to one treatment over another

Note:[a] : N given is number of included studies in the combined systematic reviews; [b] : excluded studies investigating supra-malleolar orthoses;

Table 3: Evidence table "review of systematic reviews" - studies on methods or materials

Topic	Outcomes	SR (reference)	N[a]	Findings	Future research
**Orthotic materials**	Peak pressure, contact area	Collings 2021 (14)<br>Gerrard 2020 (47)	10	- There is not a sufficiently robust evidence base to inform the selection approach regarding material combination or thickness for the best offloading.<br>- Dual or multi-density materials are frequently used, but hardly directly compared; and when done so, findings are inconsistent.<br>- Compared to shoe alone, polyurethane, polyethylene and ethyl vinyl acetate cause substantial pressure reduction.<br>- Carbon graphite decreased contact area and should not be used for pressure reduction.	- Well-designed studies are needed, testing commonly used materials in clinically relevant populations<br>- Studies should test both immediate effects, and effects after periods of wear<br>- Differences between effects of materials used in contoured or flat states should be investigated<br>- Investigate effect of different combinations of materials with different properties
**Capturing foot morphology (3D scanning vs. traditional methods)**	Time, accuracy, reliability	Farhan 2021 (48)	5[b]	- 3D scanning requires less time compared to plaster casting.<br>- Accuracy in capturing foot morphology was comparable between 3D scanning, plaster casting, digital footprints and ink footprints.<br>- Inter- and intra-rater reliability was generally comparable between 3D scanning, plaster casting and using a foam box, although some differences were found	- Evidence is insufficient, more and higher-quality research is needed<br>- User training and support, as well as experience of participants, is essential, and should both be taken into account and investigated in more detail<br>- Comparison of different 3D scanners with each other is lacking<br>- No studies on capturing foot morphology for footwear/orthoses manufacturing in children

Note: [a] : N given is number of included studies in the combined systematic reviews; [b] : the SR included 6 papers, of which 5 concerning footwear

References

1. Zafar AQ, Zamani R, Akrami M. The effectiveness of foot orthoses in the treatment of medial knee osteoarthritis: A systematic review. Gait Posture. 2020;76:238-51.
2. Shaw KE, Charlton JM, Perry CKL, de Vries CM, Redekopp MJ, White JA, et al. The effects of shoe-worn insoles on gait biomechanics in people with knee osteoarthritis: a systematic review and meta-analysis. Br J Sports Med. 2018;52(4):238-53.
3. Healy A, Farmer S, Pandyan A, Chockalingam N. A systematic review of randomized controlled trials assessing effectiveness of prosthetic and orthotic interventions. PLoS One. 2018;13(3):e0192094.
4. Xing F, Lu B, Kuang MJ, Wang Y, Zhao YL, Zhao J, et al. A systematic review and meta-analysis into the effect of lateral wedge arch support insoles for reducing knee joint load in patients with medial knee osteoarthritis. Medicine (Baltimore). 2017;96(24):e7168.
5. Tan JM, Auhl M, Menz HB, Levinger P, Munteanu SE. The effect of Masai Barefoot Technology (MBT) footwear on lower limb biomechanics: A systematic review. Gait Posture. 2016;43:76-86.
6. Arnold JB, Wong DX, Jones RK, Hill CL, Thewlis D. Lateral Wedge Insoles for Reducing Biomechanical Risk Factors for Medial Knee Osteoarthritis Progression: A Systematic Review and Meta-Analysis. Arthritis Care Res (Hoboken). 2016;68(7):936-51.
7. Parkes MJ, Maricar N, Lunt M, LaValley MP, Jones RK, Segal NA, et al. Lateral wedge insoles as a conservative treatment for pain in patients with medial knee osteoarthritis: a meta-analysis. Jama. 2013;310(7):722-30.
8. Radzimski AO, Mündermann A, Sole G. Effect of footwear on the external knee adduction moment - A systematic review. Knee. 2012;19(3):163-75.
9. Raja K, Dewan N. Efficacy of knee braces and foot orthoses in conservative management of knee osteoarthritis: a systematic review. Am J Phys Med Rehabil. 2011;90(3):247-62.
10. Ahmed S, Barwick A, Butterworth P, Nancarrow S. Footwear and insole design features that reduce neuropathic plantar forefoot ulcer risk in people with diabetes: a systematic literature review. J Foot Ankle Res. 2020;13(1):30.
11. Alahakoon C, Fernando M, Galappaththy C, Matthews EO, Lazzarini P, Moxon JV, et al. Meta-analyses of randomized controlled trials reporting the effect of home foot temperature monitoring, patient education or offloading footwear on the incidence of diabetes-related foot ulcers. Diabet Med. 2020;37(8):1266-79.
12. Crawford F, Nicolson DJ, Amanna AE, Martin A, Gupta S, Leese GP, et al. Preventing foot ulceration in diabetes: systematic review and meta-analyses of RCT data. Diabetologia. 2020;63(1):49-64.
13. van Netten JJ, Raspovic A, Lavery LA, Monteiro-Soares M, Rasmussen A, Sacco ICN, et al. Prevention of foot ulcers in the at-risk patient with diabetes: a systematic review. Diabetes Metab Res Rev 2020;36 Suppl 1:e3270.
14. Collings R, Freeman J, Latour JM, Paton J. Footwear and insole design features for offloading the diabetic at risk foot-A systematic review and meta-analyses. Endocrinol Diabetes Metab. 2021;4(1):e00132.
15. van Netten JJ, Sacco ICN, Lavery LA, Monteiro-Soares M, Rasmussen A, Raspovic A, et al. Treatment of modifiable risk factors for foot ulceration in persons with diabetes: a systematic review. Diabetes Metab Res Rev 2020;36 Suppl 1:e3271.

16. Horstink KA, van der Woude LHV, Hijmans JM. Effects of offloading devices on static and dynamic balance in patients with diabetic peripheral neuropathy: A systematic review. Rev Endocr Metab Disord. 2021.
17. Paton J, Hatton AL, Rome K, Kent B. Effects of foot and ankle devices on balance, gait and falls in adults with sensory perception loss: a systematic review. JBI Database System Rev Implement Rep. 2016;14(12):127-62.
18. Jarl G, Lundqvist LO. Adherence to wearing therapeutic shoes among people with diabetes: a systematic review and reflections. Patient Prefer Adherence. 2016;10:1521-8.
19. Swinnen E, Kerckhofs E. Compliance of patients wearing an orthotic device or orthopedic shoes: A systematic review. J Bodyw Mov Ther. 2015;19(4):759-70.
20. Tenten-Diepenmaat M, Dekker J, Heymans MW, Roorda LD, Vliet Vlieland TPM, van der Leeden M. Systematic review on the comparative effectiveness of foot orthoses in patients with rheumatoid arthritis. J Foot Ankle Res. 2019;12:32.
21. Arias-Martín I, Reina-Bueno M, Munuera-Martínez PV. Effectiveness of custom-made foot orthoses for treating forefoot pain: a systematic review. Int Orthop. 2018;42(8):1865-75.
22. Gijon-Nogueron G, Ramos-Petersen L, Ortega-Avila AB, Morales-Asencio JM, Garcia-Mayor S. Effectiveness of foot orthoses in patients with rheumatoid arthritis related to disability and pain: a systematic review and meta-analysis. Qual Life Res. 2018;27(12):3059-69.
23. Tenten-Diepenmaat M, van der Leeden M, Vliet Vlieland TPM, Roorda LD, Dekker J. The effectiveness of therapeutic shoes in patients with rheumatoid arthritis: a systematic review and meta-analysis. Rheumatol Int. 2018;38(5):749-62.
24. Conceição CS, Gomes Neto M, Mendes SM, Sá KN, Baptista AF. Systematic review and meta-analysis of effects of foot orthoses on pain and disability in rheumatoid arthritis patients. Disabil Rehabil. 2015;37(14):1209-13.
25. Hennessy K, Woodburn J, Steultjens MP. Custom foot orthoses for rheumatoid arthritis: A systematic review. Arthritis Care Res (Hoboken). 2012;64(3):311-20.
26. Schuitema D, Greve C, Postema K, Dekker R, Hijmans JM. Effectiveness of Mechanical Treatment for Plantar Fasciitis: A Systematic Review. J Sport Rehabil. 2019:1-18.
27. Gómez-Jurado I, Juárez-Jiménez JM, Munuera-Martínez PV. Orthotic treatment for stage I and II posterior tibial tendon dysfunction (flat foot): A systematic review. Clin Rehabil. 2020:269215520960121.
28. Desmyttere G, Hajizadeh M, Bleau J, Begon M. Effect of foot orthosis design on lower limb joint kinematics and kinetics during walking in flexible pes planovalgus: A systematic review and meta-analysis. Clin Biomech (Bristol, Avon). 2018;59:117-29.
29. Banwell HA, Mackintosh S, Thewlis D. Foot orthoses for adults with flexible pes planus: a systematic review. J Foot Ankle Res. 2014;7(1):23.
30. Babatunde OO, Legha A, Littlewood C, Chesterton LS, Thomas MJ, Menz HB, et al. Comparative effectiveness of treatment options for plantar heel pain: a systematic review with network meta-analysis. Br J Sports Med. 2019;53(3):182-94.
31. Rasenberg N, Riel H, Rathleff MS, Bierma-Zeinstra SMA, van Middelkoop M. Efficacy of foot orthoses for the treatment of plantar heel pain: a systematic review and meta-analysis. Br J Sports Med. 2018;52(16):1040-6.
32. Whittaker GA, Munteanu SE, Menz HB, Tan JM, Rabusin CL, Landorf KB. Foot orthoses for plantar heel pain: a systematic review and meta-analysis. Br J Sports Med. 2018;52(5):322-8.

33. Kong L, Zhou X, Huang Q, Zhu Q, Zheng Y, Tang C, et al. The effects of shoes and insoles for low back pain: a systematic review and meta-analysis of randomized controlled trials. Res Sports Med. 2020;28(4):572-87.
34. Chuter V, Spink M, Searle A, Ho A. The effectiveness of shoe insoles for the prevention and treatment of low back pain: a systematic review and meta-analysis of randomised controlled trials. BMC Musculoskeletal Disord. 2014;15:140.
35. Campbell TM, Ghaedi BB, Tanjong Ghogomu E, Welch V. Shoe Lifts for Leg Length Discrepancy in Adults With Common Painful Musculoskeletal Conditions: A Systematic Review of the Literature. Arch Phys Med Rehabil. 2018;99(5):981-93.e2.
36. Alfuth M. Textured and stimulating insoles for balance and gait impairments in patients with multiple sclerosis and Parkinson's disease: A systematic review and meta-analysis. Gait Posture. 2017;51:132-41.
37. Hurn SE, Matthews BG, Munteanu SE, Menz HB. Effectiveness of non-surgical interventions for hallux valgus: a systematic review and meta-analysis. Arthritis Care Res (Hoboken). 2021.
38. Scott LA, Munteanu SE, Menz HB. Effectiveness of orthotic devices in the treatment of Achilles tendinopathy: a systematic review. Sports Med. 2015;45(1):95-110.
39. Collins NJ, Bisset LM, Crossley KM, Vicenzino B. Efficacy of nonsurgical interventions for anterior knee pain: systematic review and meta-analysis of randomized trials. Sports Med. 2012;42(1):31-49.
40. Choi JY, Hong WH, Suh JS, Han JH, Lee DJ, Lee YJ. The long-term structural effect of orthoses for pediatric flexible flat foot: A systematic review. Foot Ankle Surg. 2020;26(2):181-8.
41. Hill M, Healy A, Chockalingam N. Effectiveness of therapeutic footwear for children: A systematic review. J Foot Ankle Res. 2020;13(1):23.
42. Dars S, Uden H, Banwell HA, Kumar S. The effectiveness of non-surgical intervention (Foot Orthoses) for paediatric flexible pes planus: A systematic review: Update. PLoS One. 2018;13(2):e0193060.
43. Paleg G, Romness M, Livingstone R. Interventions to improve sensory and motor outcomes for young children with central hypotonia: A systematic review. J Pediatr Rehabil Med. 2018;11(1):57-70.
44. Weber A, Martin K. Efficacy of orthoses for children with hypotonia: a systematic review. Pediatr Phys Ther. 2014;26(1):38-47.
45. Fellas A, Coda A, Hawke F. Physical and Mechanical Therapies for Lower-Limb Problems in Juvenile Idiopathic Arthritis(A Systematic Review with Meta-Analysis). J Am Podiatr Med Assoc. 2017;107(5):399-412.
46. James AM, Williams CM, Haines TP. "Effectiveness of interventions in reducing pain and maintaining physical activity in children and adolescents with calcaneal apophysitis (Sever's disease): a systematic review". J Foot Ankle Res. 2013;6(1):16.
47. Gerrard JM, Bonanno DR, Whittaker GA, Landorf KB. Effect of different orthotic materials on plantar pressures: a systematic review. J Foot Ankle Res. 2020;13(1):35.
48. Farhan M, Wang JZ, Bray P, Burns J, Cheng TL. Comparison of 3D scanning versus traditional methods of capturing foot and ankle morphology for the fabrication of orthoses: a systematic review. J Foot Ankle Res. 2021;14(1):2.
