# Supplementary material for: Development of a research agenda for medical grade footwear in the Netherlands: A multidisciplinary multiphase project to determine the key research questions to advance scientific knowledge in the field
Source: J Foot Ankle Res. 2024 Jul 2;17(3):e12016. doi: 10.1002/jfa2.12016 (PMC11633342; doi:10.1002/jfa2.12016)
Supplement: Supplementary file 3 — Supporting Information S3 [file JFA2-17-e12016-s002.pdf]

Additional File 3 – part of the manuscript "Development of a research agenda for medical grade footwear: a multidisciplinary multiphase project to determine the key research questions to advance scientific knowledge in the field"

Additional file 3: List of research questions submitted for prioritization

In this additional file, the 65 research questions that were submitted for prioritization in Phase 5 are shown. The research questions are shown in one table for each of the five domains included in phase 5.

The research questions are ordered based on the priority score based on the scores of all participants (second column), from highest to lowest. Priority scores of professionals only (third column) and users only (fourth column) are also shown. Shaded cells indicate that this research question was in the top-3 within that domain, within that (sub)group.

The priority score was calculated as the percentage of participants who indicated that specific research question as a priority (numerator) and the total number of valid responses (denominator).

The questions have been unilaterally translated from Dutch to English using DeepL (see Methods section in the manuscript for further details).

Table 4: Prioritization of research questions within the "Behavior and User" domain.

**Research question**	**Priority Everyone (n=152)**	**Professionals (n=138)**	**Users (n=14)**
What factors influence the use and usability of orthopedic footwear?	**37% n=56**	**36% n=49**	**50% n=7**
Does multidisciplinary prescribing and delivery of orthopedic footwear lead to increased utilization and better usability?	**36% n=54**	**37% n=51**	21% n=3
To what extent can the acceptance of orthopedic shoes be promoted in people who have difficulty with them?	**35% n=53**	**36% n=50**	21% n=3
What are the expectations and wishes of people who are fitted with orthopedic footwear, and to what extent can these be kept realistic?	34% n=51	34% n=47	29% n=4
To what extent can communication and education during prescription and delivery of orthopedic footwear be improved, and does this lead to more use and better usability?	32% n=48	33% n=46	14% n=2
Does having different pairs of orthopedic footwear for different (social) situations result in more use and better usability?	26% n=40	25% n=35	**36% n=5**
How much, under what circumstances and by what patients are orthopedic footwear worn?	22% n=34	23% n=32	14% n=2
To what extent can shared decision-making be implemented in the orthopedic footwear process, and does it lead to increased utilization and improved usability?	21% n=32	20% n=28	29% n=4
To what extent can you create a standardized user profile that includes individual behavioral characteristics, and thus further personalize the prescription of orthopedic footwear?	17% n=26	17% n=23	21% n=3
What is the usability of orthopedic footwear, and how does it change during long-term use?	15% n=23	13% n=18	**36% n=5**
What does personalized communication with a user around prescription, fitting, delivery and follow-up of orthopedic footwear look like?	11% n=17	12% n=17	0% n=0

Table 5: Prioritization of research questions within the "Clinical Effectiveness" domain.

**Research question**	**Priority Everyone (n=150)**	**Professionals (n=136)**	**Users (n=14)**
What is the cost-effectiveness of in-shoe pressure measurement-optimized orthopedic shoe devices in patients with sensory impairment without a previous plantar diabetic foot ulcer?	**50% n=74**	**48% n=65**	**64% n=9**
What are appropriate uniform clinical and patient-reported outcome measures of orthopedic footwear to demonstrate effectiveness and monitor nationwide?	**39% n=59**	**38% n=51**	**57% n=8**
What is the cost-effectiveness of provisional orthopedic footwear compared to casts in the treatment of plantar diabetic foot ulcer?	**36% n=53**	**37% n=50**	21% n=3
What is the effectiveness of orthopedic footwear devices in people at increased risk of falling?	32% n=48	32% n=43	**36% n=5**
What is the difference in effectiveness between orthotics constructed from the corrected foot shape and orthotics constructed from an insole with additional elements applied to it?	26% n=38	25% n=34	29% n=4
In people with flaccid paresis, what is the most appropriate height, rigidity and placement (loose or built-in) of a peroneal nerve device in orthopedic footwear?	26% n=38	27% n=36	14% n=2
What is the effectiveness of corrective shoe devices compared to non-intervention in children with pes planus?	22% n=32	24% n=32	0% n=0
In children with idiopathic toe gait or mild cerebral palsy, is an orthopedic shoe with sole suspension more effective than an ankle-foot orthosis?	17% n=25	19% n=25	0% n=0
What is the effectiveness of orthopedic footwear in people with lower leg paresis who cannot use an ankle-foot orthosis?	13% n=20	15% n=20	0% n=0
What is the most appropriate time and type of orthopedic shoe provision in children with hypotonia to achieve effective improvement in gait and biomechanics?	9% n=13	10% n=13	0% n=0
In children with hypotonia, is an orthopedic shoe fitting more effective than an ankle-foot orthosis?	7% n=11	8% n=11	0% n=0

Table 6: Prioritization of research questions within the "Technical Effectiveness" domain.

Research question	Priority Everyone (n=144)	Professionals (n=130)	Users (n=14)
What is the effect of wearing orthopedic footwear on muscle strength in the foot and lower leg?	**32% n=46**	**30% n=39**	**50% n=7**
What is the difference in effectiveness between a shank reinforcement at the supplement or between the lining and over leather on biomechanics and usability?	**31% n=45**	**33% n=43**	14% n=2
What is difference between loaded and unloaded fitting of orthopedic footwear on biomechanics?	**31% n=45**	**34% n=44**	7% n=1
What is the effect of different types of settlement in orthopedic footwear (round, two-phase, polyphase) on biomechanics?	29% n=41	29% n=37	**29% n=4**
How do shaft height and flexible or rigid ankle closures affect biomechanics?	26% n=37	25% n=32	**36% n=5**
What is the long-term effect of different shoe devices (settlement, materials and hardness) on biomechanics in people with diabetes?	24% n=34	21% n=27	50% n=7
What is the effect of a sole offset in the supplement/sole versus one in the shoe on biomechanics?	20% n=29	22% n=29	0% n=0
How does the weight of orthopedic footwear affect biomechanics and usability in people with muscle disease?	19% n=27	19% n=25	14% n=2
What is the effect of heel lift height on biomechanics?	17% n=25	19% n=24	7% n=14
What are contraindications for sole stiffening in orthopedic footwear in people with osteoarthritis-related pain?	17% n=25	19% n=25	0% n=0
What is the difference in biomechanical effect of the same orthotic worn in different (types of) shoes?	12% n=17	12% n=15	14% n=2
What is the effect of a dorsiflexion angle less than 90 degrees imposed by orthopedic footwear in people with spasm?	8% n=12	9% n=12	0% n=0
What is the effect of different types of heels in orthopedic footwear on biomechanics?	6% n=9	7% n=9	0% n=0
How do different types of shoe closures affect usability for people with half-sided paralysis?	6% n=9	7% n=9	0% n=0

Table 7: Prioritization of research questions within the "Processes" domain.

**Research question**	**Priority Everyone (n=139)**	**Professionals (n=126)**	**Users (n=13)**
What is the most appropriate time to move from cast or other pressure relief treatment of a diabetic foot ulcer to orthopedic footwear?	**36% n=50**	**37% n=47**	23% n=3
Which indication or condition fits which type of orthopedic shoe facility and when do you switch to another facility (confection, OVAC, OSB, OSB modular, OSA low, OSA high, EVO)?	**36% n=50**	**39% n=49**	8% n=1
What is the effect of a trial shoe on use and usability of the final orthopedic footwear?	**33% n=46**	**35% n=44**	15% n=2
To what extent can the request for help and foot diagnostics be objectified, and does that lead to more uniformity in prescribing?	27% n=38	29% n=36	15% n=2
What factors determine the wear and life span of orthopedic footwear?	20% n=28	16% n=20	**62% n=8**
What is the cost-effectiveness of orthopedic footwear for which many services are provided in terms of evaluation and aftercare?	17% n=23	17% n=21	15% n=2
What is the use and effectiveness of existing protocols, algorithms and portals for shoe prescription?	17% n=23	18% n=23	0% n=0
What are supporting and hindering factors in the use of stepped care in the prescription of orthopedic footwear?	17% n=23	18% n=23	0% n=0
To what extent can the complexity of a request for help for an orthopedic shoe facility be expressed in 1 score?	16% n=22	14% n=18	**31% n=4**
What is the association between frequency of checking after delivery of orthopedic footwear and use, serviceability, wear and clinical outcomes?	15% n=21	11% n=14	**54% n=7**
To what extent can the "Indication Portal" be completed as reliably as possible to make central registration of all prescribed orthopedic footwear available in the Netherlands?	15% n=21	17% n=21	0% n=0
To what extent can a repeat pair be fitted and delivered remotely via telemedicine in a patient where demand for help and foot are unchanged?	13% n=18	13% n=16	15% n=2
What are supporting and hindering factors in prescribing and fitting modular orthopedic footwear?	9% n=13	10% n=13	0% n=0
To what extent do delays and errors in the process of delivering orthopedic footwear occur and what are the consequences?	7% n=10	6% n=8	15% n=2

Table 8: Prioritization of research questions within the "Innovations" domain.

**Research question**	**Priority**<br>**Everyone (n=138)**	**Professionals (n=125)**	**Users (n=13)**
To what extent can orthopedic summer footwear be developed that has the same effectiveness as regular orthopedic footwear, but is more ventilated to combat heat and sweaty feet?	**33%**<br>**n=45**	29%<br>n=36	**70%**<br>**n=9**
To what extent can new materials or devices be used to make lighter but equally effective orthopedic footwear?	**33%**<br>**n=46**	**33%**<br>**n=42**	**31%**<br>**n=4**
What is the difference between digital fitting and fitting with plaster cast on the effectiveness, usability and cost of orthopedic footwear?	**33%**<br>**n=45**	**34%**<br>**n=43**	15%<br>n=2
To what extent can the appearance of an individual orthopedic shoe be digitally visualized to show in advance to the user, and does that lead to better expectations, acceptance and use?	30%<br>n=42	**34%**<br>**n=42**	0%<br>n=0
To what extent can a measurement tool be developed to measure shear forces in the shoe?	25%<br>n=35	26%<br>n=33	15%<br>n=2
What are the uses and effects of new materials and manufacturing techniques of orthopedic shoe devices, such as 3D printing, compared to more traditional techniques and materials?	22%<br>n=30	22%<br>n=28	15%<br>n=2
To what extent can a foot pressure measurement during full load be integrated during fitting, and does it lead to a better last?	12%<br>n=17	11%<br>n=14	23%<br>n=3
To what extent can pressure on the non-plantar side of the foot be reliably measured?	15%<br>n=20	14%<br>n=18	15%<br>n=2
To what extent can an OSB home shoe be developed that has the same effectiveness as regular OSB but is more usable and increases usage?	15%<br>n=20	16%<br>n=20	0%<br>n=0
To what extent can self-adaptive (smart) orthopedic footwear be made that adapts to use, pressure, walking speed, etc?	14%<br>n=19	14%<br>n=17	15%<br>n=2
To what extent can orthopedic safety footwear be made that is light and can also be adjusted after delivery?	14%<br>n=19	15%<br>n=19	0%<br>n=0
To what extent do the outcomes of different in-shoe foot pressure measurement systems differ from each other, and can this be corrected so that the outcomes become comparable or generic?	11%<br>n=15	12%<br>n=15	0%<br>n=0
To what extent can a sock be developed that provides significant pressure relief under the foot?	10%<br>n=14	6%<br>n=8	**46%**<br>**n=6**
What is the 3D foot scanner with the best value for money for orthopedic footwear fitting?	9%<br>n=13	10%<br>n=12	8%<br>n=1
What is the in-shoe foot pressure measurement system with the best value for money for evaluating orthopedic footwear?	7%<br>n=9	6%<br>n=7	15%<br>n=2
